# The Location of Substitutions and Bacterial Genome Arrangements

**DOI:** 10.1093/gbe/evaa260

**Published:** 2020-12-15

**Authors:** Daniella F Lato, G Brian Golding

**Affiliations:** Department of Biology, McMaster University, Hamilton, Ontario, Canada

**Keywords:** genome location, substitution, genomic structure, origin of replication, bacteria

## Abstract

Increasing evidence supports the notion that different regions of a genome have unique rates of molecular change. This variation is particularly evident in bacterial genomes where previous studies have reported gene expression and essentiality tend to decrease, whereas substitution rates usually increase with increasing distance from the origin of replication. Genomic reorganization such as rearrangements occur frequently in bacteria and allow for the introduction and restructuring of genetic content, creating gradients of molecular traits along genomes. Here, we explore the interplay of these phenomena by mapping substitutions to the genomes of *Escherichia coli*, *Bacillus subtilis*, *Streptomyces*, and *Sinorhizobium meliloti*, quantifying how many substitutions have occurred at each position in the genome. Preceding work indicates that substitution rate significantly increases with distance from the origin. Using a larger sample size and accounting for genome rearrangements through ancestral reconstruction, our analysis demonstrates that the correlation between the number of substitutions and the distance from the origin of replication is significant but small and inconsistent in direction. Some replicons had a significantly decreasing trend (*E. coli* and the chromosome of *S. meliloti*), whereas others showed the opposite significant trend (*B. subtilis*, *Streptomyces*, pSymA and pSymB in *S. meliloti*). d*N*, d*S*, and *ω* were examined across all genes and there was no significant correlation between those values and distance from the origin. This study highlights the impact that genomic rearrangements and location have on molecular trends in some bacteria, illustrating the importance of considering spatial trends in molecular evolutionary analysis. Assuming that molecular trends are exclusively in one direction can be problematic.

**Significance**

Previous studies have demonstrated that genomic position in bacterial genomes impacts many molecular trends such as gene expression and substitution rate. However, these studies have failed to incorporate information about genomic reorganization, such as rearrangements, into their analysis and often used few taxa. Using ancestral reconstruction to account for genomic reorganization, we have found that the number of substitutions significantly changes depending on bacterial genomic position. Utilizing information about genomic rearrangements, we demonstrate that although most individual correlations between the number of substitutions and distance from the origin of replication are significant, the values are small and inconsistent in direction. Consequently, varying substitution trends are detected when considering all bacterial species in this analysis.

## Introduction

Bacterial genomes are subject to the introduction and reorganization of genetic information through processes such as horizontal gene transfer (HGT), rearrangements, duplications, and inversions. These processes happen frequently and are important sources of genomic variation (Ochman et al. 2000; Epstein et al. 2014). Over a long-term evolutionary experiment (25 years), it has been observed that there can be anywhere between 5 and 20 rearrangement events within a single lineage (identified from each population after 40,000 generations) (Raeside et al. 2014), and some of these spontaneous rearrangements (20–40%) persist in bacterial populations (Sun et al. 2012). DNA that is acquired through HGT or other genomic rearrangements can come from the same and/or different species of bacteria, allowing useful genes to be integrated into new genomes (Ochman et al. 2000). Genomic reorganization, such as rearrangements, duplications, and inversions, provide bacteria with the opportunity to fine tune existing gene expression, dosage, and replication. Bacteria cannot escape genome reorganizations, and therefore incorporating past reorganization is a crucial component of bacterial evolutionary analyses and can be done through multigenome alignment programs, such as progressiveMauve (Darling et al. 2010), which are rearrangement aware.

Changes in the genomic structure of a bacterial genome may provide new genomic landscapes capable of altering gene regulation. Here we will consider three main types of bacterial genomic structures: circular chromosomes, linear chromosomes, and multirepliconic genomes. Secondary replicons of multirepliconic bacteria are hypothesized to predominantly contain niche-specific genes (Heidelberg et al. 2000; Egan et al. 2005). These replicons generally contain genes that have distinctive rates of evolution and selection acting upon them (Heidelberg et al. 2000). This allows the bacteria to thrive in rapidly changing environments, with varying molecular traits associated with each replicon (Heidelberg et al. 2000; Cooper et al. 2010; Morrow and Cooper 2012; Galardini et al. 2013; Jiao et al. 2018).

A previous multipartite genome investigation with four genomes of *Burkholderia* has shown that the primary chromosome is highly conserved and has higher gene expression compared with the secondary replicons which are less conserved (Morrow and Cooper 2012). A similar study using a minimum of four genomes from *Burkholderia*, *Vibrio*, *Xanthomonas*, and *Bordetella* also discovered that the primary chromosomes are conserved, with higher gene expression compared with the secondary replicons (Cooper et al. 2010). However, molecular differences between secondary replicons vary between bacterial species. In *S. meliloti*, pSymB appears to be more transcriptionally integrated with the chromosome compared with pSymA and this could be a function of the difference in evolutionary time passed, with pSymB being older than pSymA, and the amount of gene flow between these secondary replicons (DiCenzo et al. 2018). Additionally, primary chromosomes typically have lower substitution (Morrow and Cooper 2012) and evolutionary rates (Cooper et al. 2010) compared with the secondary replicons. Housekeeping genes usually reside on the primary chromosome, and the secondary replicons usually contain parts of the accessory genome, which could account for the substitution and evolutionary rate differences between primary and secondary replicons (Cooper et al. 2010; Flynn et al. 2010; Morrow and Cooper 2012; Jiao et al. 2018). It has been suggested that the differences in gene content between replicons of multirepliconic bacteria may be due to delays in replication (Flynn et al. 2010; Morrow and Cooper 2012). To maintain synchronization, due to the offset of different sequence lengths between primary and secondary replicons, the secondary replicons begin replication after the primary chromosome (Flynn et al. 2010; Morrow and Cooper 2012).

Prior research on molecular trends when moving from the origin of replication to the terminus have determined that gene expression is increased near the origin (Couturier and Rocha 2006; Kosmidis et al. 2020; Lato and Golding 2020), and genes become less conserved with increasing distance from the origin (Rocha and Danchin 2004; Couturier and Rocha 2006). Analyses with a few bacterial species have replicated these results and found that gene expression decreases with increasing distance from the origin (*Burkholderia*; Morrow and Cooper 2012) and substitution rates (nonsynonymous (d*N*), synonymous (d*S*)) and their ratio (d*N*/d*S*) increase with distance from the origin of replication (*Burkholderia*, *Vibrio*, *Bordetella*, *Xanthomonas*; Cooper et al. 2010: *Burkholderia*; Morrow and Cooper 2012). It is speculated that genes near the terminus are more prone to recombination, whereas genes near the origin have a higher prevalence of recombination repair (Sharp et al. 1989; Flynn et al. 2010). Genes near the terminus therefore often have more variation and are less conserved compared with those near the origin of replication (Sharp et al. 1989; Flynn et al. 2010). Additionally, genes found within the core genome are typically located near the origin of replication, whereas genes associated with the accessory genome are found near the terminus (Couturier and Rocha 2006; Flynn et al. 2010). The placement of these two gene categories may explain why near the origin, gene expression and essentiality are high (Couturier and Rocha 2006; Kosmidis et al. 2020; Lato and Golding 2020) and substitution rate is low (Flynn et al. 2010).

It is well known that substitutions and mutations have a nonrandom distribution around the genome which varies by gene and organism (Sharp et al. 1989; Cooper et al. 2010; Flynn et al. 2010; Morrow and Cooper 2012; Dillon et al. 2015). But, not all studies have a clear positive correlation with distance from the origin of replication and mutation rate. Some studies found no correlation between distance from the origin of replication and the frequencies of mutations, but they did find mutation rate to vary with position along the *Escherichia coli* chromosome (Juurik et al. 2012; Martina et al. 2012). Other investigations found no positive correlation with mutation rates and distance from the origin of replication and instead found that intermediate positions had a higher nonsynonymous mutation rate than positions farther from the origin in *E. coli* (Ochman 2003) and *Salmonella enterica* (Hudson et al. 2002; Ochman 2003). With respect to multirepliconic bacteria, some studies have found a lack of positive correlation between mutation rate and distance from the origin of replication. Dillon et al. (2015) found that base-substitution mutation rates are highest on the primary chromosomes and not the secondary replicons in *Burkholderia*, opposing previous observed evolutionary rates in work by Cooper et al. (2010). This appeared to have no relationship to the differences in nucleotide composition of these replicons, but rather due to some types of substitutions occurring at higher rates on particular replicons (Dillon et al. 2015). In a more recent study, Dillon et al. (2018), found that base-substitution mutation rates vary in a wave-like pattern in *Burkholderia* and *Vibrio*, where concurrently replicated segments have similar rates. This wave-like pattern in mutations was also seen in *E. coli* (Long et al. 2016) and mutation rates in *Pseudomonas aeruginosa* (Dettman et al. 2016). A similar wave-like pattern in base pair substitutions has been observed in *E. coli* (Foster et al. 2013; Niccum et al. 2019). The wave-like patterns are thought to be related to cell cycle functions and not sequence composition (Dillon et al. 2018). Interestingly, there are noteworthy differences in the location of the core and accessory genomes in some bacterial species. In the *Rhodobacteraceae* family, some species have core genes concentrated near the terminus, not the origin of replication (Kopejtka et al. 2019). Other species of this family have a mosaic pattern of core genes dispersed throughout the genome (Kopejtka et al. 2019). It is speculated that other factors such as HGT, phage insertion, and replication may be responsible for the conflicting placement of core genes in various *Rhodobacteraceae* species (Kopejtka et al. 2019). All of these exceptions to the previously established molecular trends raise questions about how universal these trends are.

There are a number of additional factors that are dependent on distance from the origin such as transposon insertion events (Gerdes et al. 2003), gene order (Mackiewicz et al. 2001), number of replication forks (Couturier and Rocha 2006), and nucleotide composition (Mackiewicz et al. 1999; Karlin 2001). These phenomena are also important to consider when analyzing molecular trends with respect to distance from the origin of replication.

The majority of these studies used an average of three genomes per bacteria analyzed (Couturier and Rocha 2006; Cooper et al. 2010; Flynn et al. 2010; Morrow and Cooper 2012) and failed to analyze secondary replicons of multipartite genomes (Couturier and Rocha 2006; Flynn et al. 2010). In this study, we examine the spatial substitution trends in *E. coli* (six genomes), *Bacillus subtilis* (seven genomes), *Streptomyces* (five genomes), and *Sinorhizobium meliloti* (six genomes). These bacteria contain genomic structures that range from single circular chromosomes (*E. coli* and *B. subtilis*), a linear chromosome (*Streptomyces*), and a multirepliconic genome (*S. meliloti*). This selection of bacterial taxa provides a sample that covers broad lifestyles as well as representing a number of divergent phylogentic lineages, providing a diverse sample to determine if the number of substitutions increases with increasing distance from the origin of replication. This study aims to determine what spatial substitution trends appear in these bacterial genomes when including the effects of genomic reorganization. We use the ancestral states of substitutions and the ancestral genomic positions of the substitutions, leading to a more accurate estimation of multiple substitutions and genomic position. Supplemental analysis on selection patterns was also performed to elucidate the potential influences on the substitution trends. We show here that the correlation between the number of substitutions and distance from the origin of replication is significant but small and inconsistent for the genomes we studied. For the majority of the replicons investigated, the number of substitutions increased when moving away from the origin of replication toward the terminus. But exceptions were the chromosomes of *E. coli* and *S. meliloti*, where the number of substitutions decreased with increasing distance from the origin. We did not find consistent significant correlations between d*N*, d*S*, and *ω* values and distance from the origin of replication. Possible causes and consequences of these patterns are discussed.

## Materials and Methods

A complete list of version numbers and build dates for all the programs used in this analysis can be found in supplementary table S1, Supplementary Material online, available on GitHub (www.github.com/dlato/Location_of_Substitutions_and_Bacterial_Arrangements).

### Sequence Data

Whole genomes of different strains of *E. coli*, *B. subtilis*, and *S. meliloti*, as well as various species of *Streptomyces* were downloaded from NCBI. Access date and accession numbers are given in supplementary table S2, Supplementary Material online. These bacteria inhabit a variety of different habitats and have contrasting genomic structures, providing a well-rounded sample for this analysis. Although *E. coli*, *B. subtilis*, and *Streptomyces* contain small plasmids, they are not considered multirepliconic bacteria and therefore their plasmids were not included in this analysis. *Sinorhizobium meliloti* is a multirepliconic bacterium and its two large secondary replicons were included in the analysis (pSymA and pSymB). The replicons of *S. meliloti* are known to differ in genetic content, and therefore, all analyses were performed on each individual replicon of *S. meliloti*. The genomes used for each species consisted of as many reference genomes as were practically possible (supplementary table S2, Supplementary Material online).

### Sequence Alignment

Alignments of each bacterial replicon were performed using progressiveMauve (default parameters) (Darling et al. 2010) to group the sequences of the replicons into locally colinear blocks (LCBs). This method allows for rearrangements, duplications and inversions to be taken into account. A LCB is frequently found at different genomic positions in each of the taxa analyzed. progressiveMauve defines these segments of sequence as minimally being similar between at least two of the taxa, but not necessarily between all of them. To obtain accurate information for subsequent analysis, only the subset of LCBs that were present in all taxa were considered. Each LCB was then realigned with MAFFT (-auto) (Katoh et al. 2002) to obtain a more accurate local alignment. Although progressiveMauve is good at identifying large scale rearrangements and inversions, it sometimes determined LCBs that were very small and contained questionably homologous or excessively gapped sequences (see supplementary file for more information and examples, Supplementary Material online). As a result, we used trimAl (Capella-Gutiérrez et al. 2009) to remove poorly aligned regions, which were defined as having poor homology and/or excessive gaps. We used the -strictplus setting in trimAl to automatically determine regions of unacceptable alignment.

A custom Python script was created to ensure that within each alignment LCB, the correct coding frame was present. Codon position information was obtained for each base pair in the LCBs from the GenBank file for each taxon. Each column of the alignment was only kept if all taxa had the same codon position (1, 2, or 3). Alignment columns where the codon positions were not the same were removed from the analysis.

We found that using these alignments, trimming criteria effectively removed portions of the alignment that had poor homology or were gaped. We imposed an additional minimum ungapped alignment length of 100 bp to each of the gene segments. We chose this number so that we could keep the maximum amount of information, while avoiding comparing potentially inaccurate and extremely short portions of a gene (<100 bp). These trimmed alignments of genes and gene segments are used for the remainder of the analysis.

There is a delicate balance between capturing large amounts of recombination, while still ensuring a comparison of homologous sequences. The more distantly related taxa are, the less similar the genetic sequences are, which in the case of progressiveMauve, results in a large number of short LCBs. A high number of LCBs results in the potential comparison of nonhomologous sequences, which would create incorrect results in any phylogenetic or evolutionary analysis. As a result, we had to limit the number sequences used in our analysis (see Supplementary Material online, for additional details, www.github.com/dlato/Location_of_Substitutions_and_Bacterial_Arrangements).

In addition, the number of sequences chosen for all bacteria was constrained by the computational time required to perform a progressiveMauve alignment. This computing time increases exponentially with additional genomes. For further information, please see Supplementary Material online, on GitHub at www.github.com/dlato/Location_of_Substitutions_and_Bacterial_Arrangementss.

### Protein-Coding Substitutions

To ensure that only homologous sequences were being compared, we are only considering the substitutions that reside in protein-coding regions of the genome. Any site where a gap or an ambiguous nucleotide was present, was removed from the analysis, and the remaining portions of the gene were separated and considered two distinct “genes.” The remainder of the analysis was done on each of these gene segments separately.

### Phylogenetic Trees

Rearrangements, duplications, and inversions happen frequently and must be considered when analyzing spatial genomic trends. Phylogenetic trees were created to trace the evolutionary history of large scale and local DNA rearrangements. These trees were used to determine the number of substitutions and record the genomic location of substitutions for each respective replicon. Whole-genome alignments both including and excluding the outgroups were performed using progressiveMauve and split up into LCBs that were realigned with MAFFT (see Sequence Alignment). Each of the LCBs specified by progressiveMauve was combined to create a single “super sequence.” RAxML was used to estimate phylogenetic trees both including (raxmlHPC-PTHREADS-SSE3 -T 20 -f a -x 12345 -o -N 100 -p 12345 -m GTRGAMMA) and excluding (raxmlHPC -f a -x 12345 -p 12345 -# 1000 -m GTRGAMMA) the outgroup. The tree topology from the phylogenetic tree including the outgroup was used to optimize the branch lengths for the phylogenetic tree excluding the outgroup (raxmlHPC -f T -t -p 12345 -m GTRGAMMA). Bootstrap values for this tree was calculated using 1000 replicates (raxmlHPC -f b -t -z -m GTRGAMMA). Phylogenetic trees with bootstrap support values can be found in the Supplementary Material online.

An SH test (Shimodaira and Hasegawa 1999; Goldman et al. 2000) was performed to determine if there was a significant difference between the super sequence and the tree topology of each LCB individually. Any LCBs that had a topology that was significantly different (at the 5% significance level) from the super sequence topology was removed from the remainder of the analysis. The SH test was performed using RAxML (raxmlHPC -f H -t -z -s -m GTRGAMMA) (Stamatakis 2014).

### Origin and Bidirectional Replication

**Fig. 1:**
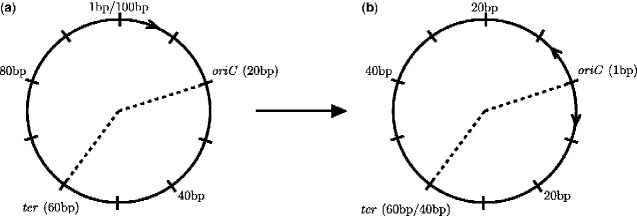
Schematic of the transformation used to scale the positions in the genome to the origin of replication and account for bidirectional replication. Circle (*a*) represents the original replicon genome without any transformation. Circle (*b*) represents the same replicon genome after the transformation. The origin of replication is denoted by *oriC* and the terminus of replication is denoted by *ter*. The dashed line represents the two halves of the replicon. The replicon genome in this example is 100 bp in length. Every 10 bp is denoted by a tick on the genome. The origin in (*a*) is at position 20 in the genome and is transformed in (*b*) to become position 1. The terminus is at position 60 in (*a*) and position 60/40 in (*b*). The terminus has two positions in (*b*) depending on which replicon half is being accounted for. If the replication half to the right of the origin is considered, the terminus will be at position 40. If the replication half to the left of the origin is considered, the terminus will be at position 60. Position 40 in (*a*) becomes position 20 in (*b*). Position 80 in (*a*) becomes position 40 in (*b*) due to the bidirectional nature of bacterial replication (Figure from: D.F. Lato and G.B. Golding. Spatial patterns of gene expression in bacterial genomes, *J Mol Evol*. published June 2020, Springer Nature).

For each bacteria, the origin of replication was denoted as the beginning of the *oriC* region for the chromosomal replicons, and the beginning of the *repC* (Pinto et al. 2011) region for the secondary replicons of *S. meliloti* (supplementary table S4, Supplementary Material online). This origin of replication position was calibrated to be the beginning of the genome, position 1, and remaining positions in the genome were all scaled around this origin of replication taking into account the bidirectional nature of bacterial replication (fig. 1).

The terminus of replication was determined using the Database of Bacterial Replication Terminus (Kono et al. 2011), which uses the prediction of *dif* sequences (normally found at the terminus), as a proxy for the location of the terminus (Clerget 1991; Blakely et al. 1993). For pSymA and pSymB of *S. meliloti*, the terminus is not listed in the database, thus the terminus location was assigned to the midpoint between the origin of replication and the end of the replicon. Replication in the linear chromosome of *Streptomyces* begins at the origin of replication, located to the right of the middle of the replicon (Heidelberg et al. 2000) and terminates at each end of the chromosome arms (Heidelberg et al. 2000) (supplementary table S4, Supplementary Material online).

We have chosen a single base to represent the origin and terminus of replication. In reality, the origin of replication is often multiple base pairs long, and there has been no evidence for site-specific termination of replication, but rather a small genomic region where replication concludes based on various other factors (Duggin and Bell 2009). To determine the effect of the exact location of the origin and terminus, permutation tests shuffling the *oriC* position by 10,000 bp increments in each direction from the original origin (supplementary table S4, Supplementary Material online) to a maximum of 100,000 bp in each direction were performed. These results showed that moving the origin of replication does not affect the results of the analysis (supplementary table S5, Supplementary Material online). Based on this supplementary test, choosing a single base to represent the origin and terminus of replication has minimal impact on the analysis.

### Ancestral Reconstruction

**Fig. 2:**
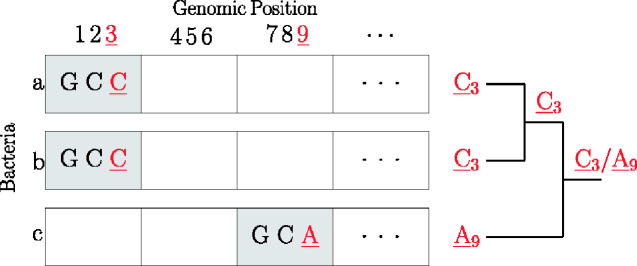
Schematic of the ancestral reconstruction of both the nucleotide and genomic position. Each horizontal row of rectangles represents three hypothetical bacterial genomes (a–c). The genomic position is indicated at the top of the diagram. The phylogenetic tree showing the relationship between all three bacteria is pictured on the right of the diagram. The light gray rectangle denotes the homologous genomic. In bacteria (a) and (b), this segment is located at genomic positions 1–3. In bacteria (c), this segment is located at genomic positions 7–9. Within this genomic region of interest, there is a substitution where the nucleotides changed from C → A, this is highlighted in red and underlined. This would mean that in bacteria (c), there was a substitution from C → A which is also associated with a genomic position of 9. This substitution is at position 3 in bacteria (a) and (b) and position 9 in bacteria (c). This is depicted by the values (C_3_) and (A_9_). The ancestral reconstruction process in this analysis can be seen at the inner nodes of the phylogenetic tree by the values (C_3_). The most parsimonious reconstruction of the sequence and associated genomic position is having the value (C_3_) present at the ancestor of bacteria (a) and (b). The ancestral node of all three bacteria would have a reconstruction of the sequence and associated genomic position of (C_3_/A_9_). In this situation, where there is a “tie” for two most parsimonious options, the option with the highest likelihood estimate would be chosen using maximum-likelihood methods (see Yang 1997 for more details).

To track genome reorganization, nucleotide substitutions and genomic positions were reconstructed in extinct ancestors. We used the PAML (Yang 1997) package of programs, with slight modification, to reconstruct genome location and substitutions in hypothetical ancestors (fig. 2).

### Nucleotide Substitutions

The baseml program (model = 0, Mgene = 0, clock = 1, fix_kappa = 0, kappa = 5, fix_alpha = 1, alpha = 0, Malpha = 0, ncatG = 5, nparK = 0, nhomo = 0, getSE = 0, RateAncestor = 2) in the PAML package (Yang 1997) was used to determine single nucleotide substitutions within each of the alignments. This program determined the ancestral state of each nucleotide in the alignment at each node in the phylogenetic tree (fig. 2). Multiple substitutions at one site were allowed and accounted for as separate substitutions. Any nucleotides, or columns, in the alignment that had at least one gap present were not used in the analysis because the baseml program inaccurately classifies substitutions when a gap is involved. These gaped positions were categorized as missing data.

### Genomic Position

Genomic reorganization was accounted for using the genome locations specified by progressiveMauve to determine the ancestral genome positions of each taxa (fig. 2). These locations were inferred for each nucleotide in the alignment.

The codeml program (CodonFreq = F3X4, clock = 0, aaDist = 0, aaRatefile = dat/jones.dat, model = 0, NSsites = 0, Mgene = 0, fix_kappa = 0, kappa = 2, fix_omega = 0, omega = 0.4, fix_alpha = 1, alpha = 0, Malpha = 0, ncatG = 8, getSE = 0, RateAncestor = 1) (Yang 1997) from the PAML package was modified to reconstruct the ancestral genome positions at each node within the phylogenetic tree (supplementary trees: S4–S9, Supplementary Material online) of each respective replicon for each position in the alignment (fig. 2).

A custom Python script (see GitHub www.github.com/dlato/Location_of_Substitutions_and_Bacterial_Arrangements) was used to associate each of the protein-coding regions with their genomic positions and determine how many ancestral and extant substitutions were found in each region. Each branch in the tree possesses information on how each nucleotide in the alignment has moved throughout the genome to the current position in each of the taxa (fig. 2). Therefore, each segment of sequence has the opportunity to be present in one position in the genome of one taxa and a completely different position in another taxa (fig. 2).

For this portion of the analysis, each genomic position was considered unique and distinct, including positions that were separated by one base pair.

We performed a supplementary analysis to determine if clustering genomic positions based on how many base pairs separate substitutions, would significantly alter the overall spatial results (see Supplementary Material online for more details). We determined that considering each genomic position to be unique and distinct or clustering the positions did not alter the results.

### Logistic Regression

The binary nature of the data is ideal for a logistic regression to determine the statistical significance of substitution and position trends at protein-coding regions of the genome in each bacterial replicon (table 2). Any subset of points outside the interquartile range were considered outliers and ignored.

**Table 2:** Logistic Regression Analysis of the Number of Substitutions Along All Protein-Coding Positions of the Genome of the Respective Bacteria Replicons

Bacteria and Replicon	Protein-Coding Sequences Coefficient Estimate
*Escherichia coli* chromosome	−2.66 × 10^−8^***
*Bacillus subtilis* chromosome	2.76 × 10^−8^***
*Streptomyces* chromosome	6.97 × 10^−8^***
*Sinorhizobium meliloti* chromosome	−6.57 × 10^−7^***
*Sinorhizobium meliloti* pSymA	2.74 × 10^−7^***
*Sinorhizobium meliloti* pSymB	1.10 × 10^−7^***

Note.—Gray colored boxes indicate a negative logistic regression coefficient estimate. All results are statistically significant. Logistic regression was calculated after the origin of replication was moved to the beginning of the genome and all subsequent positions were scaled around the origin accounting for bidirectional replication.

All results are marked with significance codes as followed:

*** *P* <0.001.

**Fig. 3:**
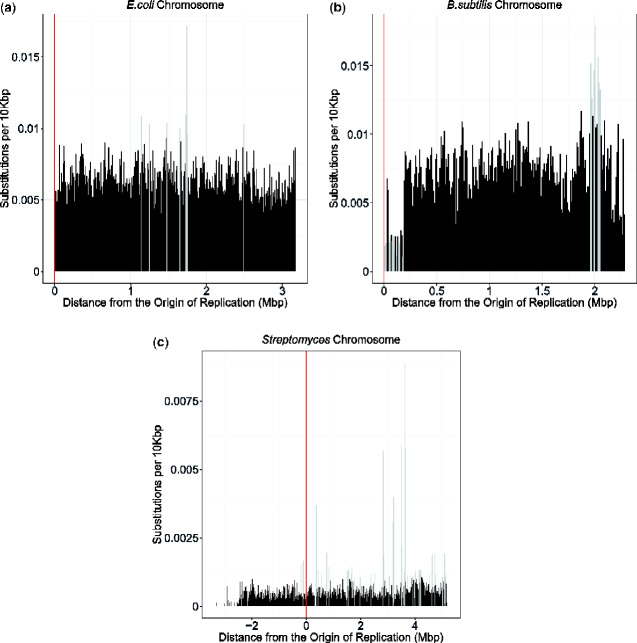
The bar graphs show the number of substitutions along the genomes of *Escherichia coli* (*a*), *Bacillus subtilis* (*b*), and *Streptomyces* (*c*). For *E. coli* and *B. subtilis*, the distance from the origin of replication is on the *x* axis beginning with the origin of replication denoted by position 0 on the left, and the terminus indicated on the far right. This distance includes the distance from the origin in both replichores. For *Streptomyces*, the origin of replication is denoted by position 0. The genome located on the shorter chromosome arm (to the left of the origin) has been given negative values, whereas the genome on the longer chromosome arm (to the right of the origin) has been given positive values. The origin of replication in the *Streptomyces* graph (*c*) has been highlighted at position 0 by a red vertical line. The *y* axis of the graphs indicate the number of substitutions per 10,000 bp found at each position of the *E. coli* (*a*), *B. subtilis* (*b*), and *Streptomyces* (*c*) genomes. Each bar represents a section of the genome that spans 10 kb. The total number of substitutions in each 10-kb region of the replicon was divided by the total number of protein-coding sites within that 10-kb region, to give the substitutions per 10 kb (*y* axis). Outliers are represented in light gray bars.

**Fig. 4:**
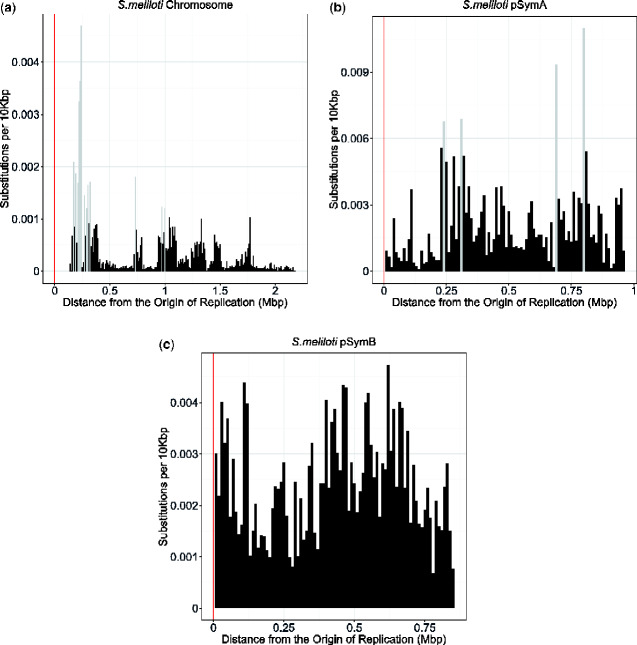
The bar graphs show the number of substitutions along the replicons of *Sinorhizobium meliloti*: chromosome (*a*), pSymA (*b*), and pSymB (*c*). Distance from the origin of replication is on the *x* axis beginning with the origin of replication denoted by position 0 on the left, and the terminus indicated on the far right. This distance includes the distance from the origin in both replichores. The *y* axis of the graph indicates the number of substitutions per 10,000 bp of the replicons of *S. meliloti*: chromosome (*a*), pSymA (*b*), and pSymB (*c*). Each bar represents a section of the genome that spans 10 kb. The total number of substitutions in each 10-kb region of the replicon was divided by the total number of protein-coding sites within that 10-kb region, to give the substitutions per 10 kb (*y* axis). Outliers are represented by light gray bars.

A visualization of substitutions in relation to distance from the origin of replication can be found in figures 3 and 4. The total number of substitutions in each 10-kb region of the replicon was divided by the total number of protein-coding sites within that 10-kb region, to give the substitutions per 10 kb (*y* axis).

### Selection

Within the protein-coding regions of the genome, we wanted to observe how selection may be acting on each of the genes in the various bacterial replicons. Calculating the synonymous (d*S*) and nonsynonymous (d*N*) substitution rates and the ratio of these two (*ω*) for each gene allows for an in-depth analysis of the selective pressures throughout the genome while accounting for genomic reorganization between the bacterial taxa. We can then relate this information to the location of the genes in the genome and determine trends between selection and distance from the origin. It has been found previously that genes closest to the origin of replication are conserved (Couturier and Rocha 2006) and tend to be a part of the core genome (Couturier and Rocha 2006; Flynn et al. 2010). We therefore expect genes closer to the origin to have fewer substitutions and therefore lower values for d*S* and d*N*.

**Fig. 5:**
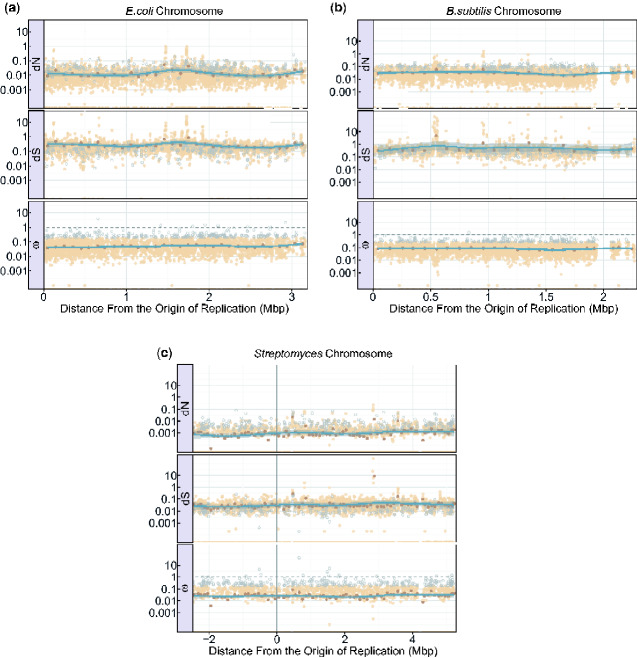
The graphs show the values of d*N*, d*S*, and *ω* along the genomes of *Escherichia coli* (*a*), *Bacillus subtilis* (*b*), and *Streptomyces* (*c*). For *E. coli* and *B. subtilis*, the distance from the origin of replication is on the *x* axis beginning with the origin of replication denoted by position 0 on the left, and the terminus indicated on the far right. For *Streptomyces*, the origin of replication is denoted by position 0. The genome located on the shorter chromosome arm (to the left of the origin) has been given negative values, whereas the genome on the longer chromosome arm (to the right of the origin) has been given positive values. The origin of replication in the *Streptomyces* graph (*c*) has been visualized at position 0 by a gray vertical line. The *y* axis of the graph indicates the value of d*N*, d*S*, and *ω* found at each gene segment position of the *E. coli* (*a*), *B. subtilis* (*b*), and *Streptomyces* (*c*) genomes. Outliers are represented by light gray open circles. The average d*N*, d*S*, and *ω* values for each 100,000 bp region of the genome was calculated and represented by the dark brown points. A trend line represented in blue (using the loess method) was fit to these average values and the associated 95% confidence intervals for this line is represented by the gray ribbon around the blue trend line. For a complete list of outlier and zero value information, please see the Supplementary Material online.

**Fig. 6:**
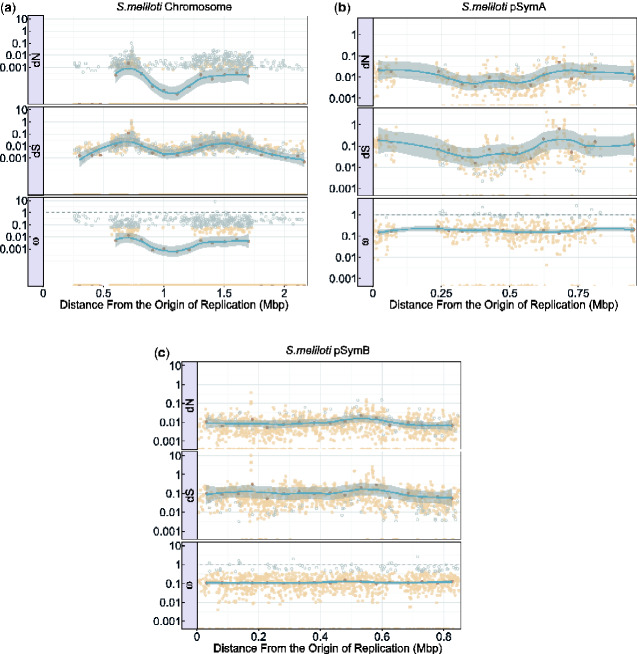
The graphs show the values of d*N*, d*S*, and *ω* along the replicons of *Sinorhizobium meliloti*, chromosome (*a*), pSymA (*b*), and pSymB (*c*). Distance from the origin of replication is on the *x* axis beginning with the origin of replication denoted by position 0 on the left, and the terminus indicated on the far right. The *y* axis of the graph indicates the value of d*N*, d*S*, and *ω* found at each gene segment position of the chromosome (*a*), pSymA (*b*), and pSymB (*c*) of *S. meliloti*. Outliers are represented by light gray open circles. The average d*N*, d*S*, and *ω* values for each 100,000 bp region (for the chromosome) and 50,000 bp region (for both pSymA and pSymB) of the replicons were calculated and represented by the dark brown points. A trend line represented in blue (using the loess method) was fit to these average values and the associated 95% confidence intervals for this line is represented by the gray ribbon around the blue trend line. For a complete list of outlier and zero value information, please see the Supplementary Material online.

The data sets used for this portion of the analysis is the same as the one used in the substitutions analysis, with the exception that we ensured all genes and gene segments of the alignment start and end with complete codons for the selection analysis (this was done through a custom Python script). Gaps or ambiguous nucleotides were also removed from these genes (Python) and are subsequently missing in the graphical representation of the distribution (figs. 5 and 6).

### Calculating d*N*, d*S*, and *ω*

The codeml program (CodonFreq = 2, clock = 0, model = 0, NSsites = 0, icode = 0, fix_omega = 0, omega = 0.4) in the PAML package (Yang 1997) was used to calculate the synonymous (d*S*) and nonsynonymous (d*N*) substitution rates and to estimate a value for *ω*. d*N*, d*S*, and *ω* were calculated on each gene/gene segment separately. The varying nucleotide models have minimal impact on the d*N* and d*S* calculations because the overall number of synonymous and nonsynonymous substitutions per site were small. There were some segments of the alignment that had poor homology (see Sequence Alignment for more information). As a result, some genes were split into multiple parts, removing those segments of poor alignment. Calculations and analysis were done separately for each of these gene “segments” for the remainder of the study.

Outliers for the selection data were determined using only the *ω* values. Any subset of *ω* points outside the interquartile range were considered outliers and ignored. The associated d*N* and d*S* values for the same gene segment of each *ω* outlier were also considered outlier values. These points were subsequently removed from the analysis. We then used the d*N*, d*S*, and *ω* values of each gene or gene segment to calculate an arithmetic average of d*N*, d*S*, and *ω* for each replicon weighted by the length of each gene or gene segment. To prevent the use of undefined *ω* values, any genes where both d*N* and d*S* or d*S* were equal to zero were removed from the weighted *ω* calculation. A summary of the average d*N* and d*S* results are found in table 3.

**Table 3:** Weighted Averages for d*S*, d*N*, and *ω* Values Calculated for Each Bacterial Replicon on a Per Genome Basis Using the Gene Length as the Weight

Bacteria and Replicon	Genome Average		
	d*S*	d*N*	*ω*
*Escherichia coli* chromosome	0.2352	0.0101	0.0445
*Bacillus subtilis* chromosome	0.4134	0.0240	0.0712
*Streptomyces* chromosome	0.0468	0.0011	0.0323
*Sinorhizobium meliloti* chromosome	0.0122	0.0002	0.0042
*Sinorhizobium meliloti* pSymA	0.0839	0.0099	0.1760
*Sinorhizobium meliloti* pSymB	0.0956	0.0085	0.1148

Note.—Arithmetic mean was calculated for the per gene averages for each bacterial replicon.

Linear regressions were performed to determine if there is any correlation between d*N*, d*S*, and *ω*, respectively, and distance from the origin of replication while accounting for bidirectional replication. All linear regression results are summarized in table 4.

**Table 4:** Linear Regression to Determine the Correlations Between d*N*, d*S*, and *ω* Values and Distance from the Origin of Replication

Bacteria and Replicon	d*N*	d*S*	*ω*
*Escherichia coli* chromosome	NS	NS	4.33 × 10^−9^*** (0.007)
*Bacillus subtilis* chromosome	−6.03 × 10^−9^*** (0.004)	NS	−6.80 × 10^−9^*** (0.004)
*Streptomyces* chromosome	1.40 × 10^−10^* (0.002)	NS	NS
*Sinorhizobium meliloti* chromosome	−1.67 × 10^−10^* (0.003)	−8.67 × 10^−9^*** (0.007)	−1.20 × 10^−9^* (0.003)
*Sinorhizobium meliloti* pSymA	NS	NS	NS
*Sinorhizobium meliloti* pSymB	NS	NS	NS

Note.—A regression was performed for each bacterial replicon with outliers removed. All results are marked with significance codes as follows:

*** *P* < 0.001

* 0.01 < 0.05, NS = >0.05. The *R*^2^ values for each estimate are in brackets.

## Results

### Average Number of Substitutions

Table 1 summarizes the average number of substitutions per base pair for each bacterial replicon. The strains of *S. meliloti* chromosomes and species of *Streptomyces* chosen for this study are more similar to each other than the strains within the other bacterial replicons. This low divergence between genomes is likely the cause for lower average number of substitutions per base pair in *Streptomyces* and the chromosome of *S. meliloti*. The smaller replicons of *S. meliloti—*pSymA and pSymB—have faster substitution rates compared with the larger chromosomal replicon of the same bacteria. This is likely due to the relative decreased divergence between strains used in the *S. meliloti* chromosome analysis. pSymB has a slightly faster substitution rate compared with pSymA. These results are consistent with the general knowledge of the gene content between the smaller replicons of *S. meliloti* and the chromosome. The smaller replicons are expected to evolve more quickly. It is curious that pSymB has a slightly higher average substitution rate compared with pSymA because pSymA has been shown to be more variable in gene content and function compared with pSymB (Galardini et al. 2013).

**Table 1:** Average Number of Protein-Coding Substitutions Calculated Per Base across All Bacterial Replicons

Bacteria and Replicon	Average Number of Substitutions Per bp
*Escherichia coli* chromosome	6.48 × 10^−3^
*Bacillus subtilis* chromosome	7.56 × 10^−3^
*Streptomyces* chromosome	4.23 × 10^−4^
*Sinorhizobium meliloti* chromosome	2.43 × 10^−4^
*Sinorhizobium meliloti* pSymA	2.03 × 10^−3^
*Sinorhizobium meliloti* pSymB	2.35 × 10^−3^

Note.—Outliers and missing data are not included in the calculation.

### Logistic Regression

The logistic regression and supporting statistical information for the substitution trends are found in table 2. The number of substitutions decreased when moving away from the origin of replication for the protein-coding regions of *E. coli* and the chromosome of *S. meliloti*. This implies that the area near the terminus of replication in these replicon sections had less substitutions than the area near the origin of replication. pSymA and pSymB of *S. meliloti*, *B. subtilis*, and *Streptomyces* showed the opposite trend from the other bacterial replicons, with a decreased number of substitutions present near the origin of replication compared with the terminus. All of the correlation estimates between the number of substitutions and distance from the origin of replication are small and vary in their sign. From these inconsistent results, we conclude that there is no consistent, significant correlation between the number of substitutions and distance from the origin of replication.

Additional tests grouping the number of substitutions in varying windows of the genomes (10, 25, 50, 100, 200, and 400 kb) were done to supplement the logistic regression results. The total number of substitutions per window size (10, 25, 50, 100, 200, and 400 kb) was totaled and a linear regression was performed on those totals and distance from the origin of replication (supplementary tables S11 and S12, Supplementary Material online). These results are inconsistent in sign when significant, mirroring the results from the logistic regression (table 2). Based on these inconsistent supplemental results, we remain confident in saying that there is no consistent, significant correlation between the number of substitutions and distance from the origin of replication.

A nonlinear analysis of the variation in the number of substitutions per 10 kb with distance from the origin of replication was performed (supplementary figs. S13–S18, Supplementary Material online). The results from this analysis complement the logistic regression results, the total number of substitutions varies with distance from the origin of replication, but the pattern and direction of this trend is inconsistent between bacterial replicons.

Additional analysis were done to ensure that the individual taxa chosen in this analysis were not influencing the overall conclusion about the distribution of substitutions along bacterial genomes. We systematically removed each taxa from the substitutions analysis (see Supplementary Material online) to determine if any particular taxa were influencing the results. These results are summarized in supplementary table S15, Supplementary Material online. From this supplemental analysis, we have come to the same conclusion that the number of substitutions significantly varies with distance from the origin of replication, but the direction of this trend is inconsistent in sign. In supplementary table S15, Supplementary Material online, when most of the taxa in each species is removed, the correlation between the number of substitutions and distance from the origin of replication is significant and follows the same sign (positive or negative) within a replicon. However, occasionally the sign of this trend flips for particular strains/species that are removed. We determined this change is due to a new “outgroup” specified in the tree (via the removal of the previous outgroup in *Streptomyces* and pSymA of *S. meliloti*), or it is likely that the taxa which was removed was the ancestral genomic position for the substitutions and when it is removed, the ancestral genomic position changes (*Bacillus subtilis* and pSymB of *S. meliloti*). A complete discussion of this can be found in the Supplementary Material online. Future work exploring the ancestral states of nucleotides and genomic position using different species/strains would be able to test for this.

Areas of the bacterial genomes in this analysis with extremely high number of substitutions per 10-kb region are regions that encode mostly small (65–150 amino acids long) hypothetical proteins (see supplementary table S10, Supplementary Material online). These regions could have higher numbers of substitutions due to the small length of these genes and unclear characterization of the associated encoded proteins.

The density of ancestral and extant substitutions in protein-coding regions across each bacterial replicon can be seen in figures 3 and 4. These figures supplement the logistic regression analysis and provide information on the frequency of substitutions in relation to the distance from the origin of replication while also taking into account the bidirectional replication (see Origin and Bidirectional Replication). Areas of these graphs that look sparse or appear to be “missing” data from some genomic regions have had data excluded in these regions because they did not meet the alignment quality and trimming requirements specified in this analysis (see Sequence Alignment).

### Selection

The distribution of d*N*, d*S*, and *ω* values across each bacterial replicon can be seen in figures 5 and 6. These figures provide information on the values of d*N*, d*S*, and *ω* in relation to the distance from the origin of replication while taking into account bidirectional replication (see Origin and Bidirectional Replication). Areas of these graphs that look sparse or appear to be missing data from some genomic regions have had data excluded in these regions because they did not meet the alignment quality and trimming requirements specified in this analysis (see Sequence Alignment). High d*S* values in figures 5 and 6 are reflective of divergent portions of a gene alignment. For a complete discussion of these values, please see the Supplementary Material online. d*N* and *ω* values of zero are produced by low numbers of substitutions, from in an overwhelming number of identical LCBs (for a complete account of zero values, please see the Supplementary Material online).

The genome average values of d*S*, d*N*, and *ω* for each replicon are found in table 3. All bacterial replicons had average per genome d*S* values that were higher than the respective d*N* values. This is as expected because most genes should be under purifying selection.

Linear regressions were performed to determine if there is any correlation between d*N*, d*S*, and *ω*, respectively, and distance from the origin of replication while accounting of bidirectional replication. All linear regression results are summarized in table 4. All values for d*N*, d*S*, and *ω*, aside from any considered outliers (see Methods), were used in the regression analysis. We were unable to find significant linear regression coefficients for the majority of the bacterial replicons used in this analysis. The sporadic significant and nonsignificant positive and negative coefficient estimates do not provide a clear picture of how substitution rates and *ω* change with distance from the origin of replication, and we therefore cannot conclude that there is one overarching spatial trend for d*N*, d*S*, or *ω* values.

Additional tests using the average d*N*, d*S*, or *ω* values in varying windows of the genomes (10, 25, 50, 100, 200, and 400 kb) were done to supplement the linear regression results done on all data points. The average d*N*, d*S*, or *ω* values per window size (10, 25, 50, 100, 200, and 400 kb) were calculated, and a linear regression was performed on those average values and distance from the origin of replication (supplementary table S18, Supplementary Material online). These results are mostly not significant and ones that are significant are inconsistent in sign, mirroring the results from the linear regression on all data points (table 4). Based on these inconsistent supplemental results, we are confident that there is no significant correlation between the value of d*N*, d*S*, or *ω* and distance from the origin of replication.

## Discussion

To date there has been a large body of work looking at how molecular trends such as gene expression (Couturier and Rocha 2006; Cooper et al. 2010; Morrow and Cooper 2012; Kosmidis et al. 2020; Lato and Golding 2020), substitution rates (Sharp et al. 1989; Cooper et al. 2010; Flynn et al. 2010; Morrow and Cooper 2012), and mutation rates (Hudson et al. 2002; Ochman 2003; Juurik et al. 2012; Martina et al. 2012; Dettman et al. 2016; Dillon et al. 2018) vary with genomic position. The general consensus is that substitution rate is highest near the terminus of replication and relatively low near the origin (Sharp et al. 1989; Cooper et al. 2010; Flynn et al. 2010; Morrow and Cooper 2012). Most of these studies used an average of three genomes per bacteria analyzed (Couturier and Rocha 2006; Flynn et al. 2010; Cooper et al. 2010; Morrow and Cooper 2012) and failed to analyze secondary replicons of multipartite genomes (Couturier and Rocha 2006; Flynn et al. 2010). However, there are also a number of studies that failed to observe this positive linear correlation in the absence of selection with mutations and mutation rates (Hudson et al. 2002; Ochman 2003; Juurik et al. 2012; Martina et al. 2012; Foster et al. 2013; Dettman et al. 2016; Long et al. 2016; Dillon et al. 2018). In this work, we explored the spatial trends of substitutions and d*N*, d*S*, and *ω* values along bacterial genomes to add to the previous knowledge of spatial trends in bacteria. This study takes a unique approach to the analysis of how the number of substitutions changes with distance from the origin of replication by accounting for local and large scale genomic rearrangements by utilizing ancestral reconstruction techniques of both substitutions and genomic positions.

Although thousands of bacterial genomes have been sequenced for bacteria with different genomic structures, the majority of these genomes are incomplete and composed of scaffolds or contigs. For this analysis, a complete genome, free of gaps or contigs, was necessary to accurately track substitutions and their genomic locations. Incomplete genomes would have gaps in genome positions, leaving missing information about substitutions for these segments of sequence. Therefore, we wished to consider only complete genomes. We would like to expand our analysis in the future to incorporate more genomes and taxa, but currently, there are few that are suitable to our specific requirements.

We were unable to observe a consistent significant correlation between distance from the origin of replication and the number of substitutions per site as well as the values of d*N*, d*S*, and *ω* in the replicons that were analyzed. This necessitates further in-depth analysis of other molecular trends in bacterial genomes while accounting for genomic reorganization. Using tools such as ancestral reconstruction and the history of rearrangements, other spatial molecular trends in bacteria can be elucidated. This can be applied to gene expression and essentiality, to determine how these molecular components are impacted by rearrangements and what this tells us about the organization of genes along bacterial genomes.

### Spatial Substitution Trends

We have demonstrated here that any correlation between the number of substitutions and genomic position in our bacterial species is significant but small and inconsistent in sign (table 2). In this analysis, we have looked at protein-coding genes within the genomes of *E. coli*, *B. subtilis*, *Streptomyces*, and *S. meliloti*, including both core and accessory genes. Previous studies looking at substitution rates and genomic position typically looked at orthologous genes with similar genomic positions (Cooper et al. 2010; Morrow and Cooper 2012). The discrepancy between our results and previously published analysis may be due to our alignments having dissimilar genomic positions in some taxa and the inclusion of genomic reorganization. Some segments of the genomes have relatively high numbers of substitutions compared with the rest of the genome. For example, the high bars located near 2 million base pairs (Mb) from the origin in *B. subtilis* (fig. 3*b*) seem to have an increase in the number of substitutions in this genomic segment relative to the other 10-kb regions. These high substitution regions are homologous genes or gene segments that happen to have amino acid changes which are driving the high number of substitutions in those bars. An illustrative example of one such gene segment can be found in the supplementary figures S11 and S12, Supplementary Material online.

The multirepliconic nature of *S. meliloti* appears to have a small effect on the overall spatial substitution trends of each replicon. For example, the opposing spatial substitution trends (table 2 and fig. 4) of different replicons in *S. meliloti* may be due to an overrepresentation of highly expressed or essential genes located on the chromosome. We found an increased number of substitutions in the smaller replicons, pSymA and pSymB, compared with the chromosome. The smaller replicons are known to display less genomic conservation than the chromosome (Cooper et al. 2010; Morrow and Cooper 2012) and have genes used for local environmental adaptation (Medini et al. 2008; DiCenzo et al. 2019), which may explain the increased number of substitutions in pSymA and pSymB, compared with the chromosome.

A number of previous studies have complementary results regarding increasing substitution trends of bacterial replicons which was found in *B. subtilis*, *Streptomyces*, and the small replicons of *S. meliloti* in this analysis. These previous studies observed gene expression (Sharp et al. 2005; Couturier and Rocha 2006; Morrow and Cooper 2012; Lato and Golding 2020) decreases, whereas substitution rate was found to increase with increasing distance from the origin of replication (Prescott and Kuempel 1972; Morrow and Cooper 2012; Galardini et al. 2013). Genes that are less essential and often expressed less tend to evolve quickly compared with more conserved genes with higher expression levels (Sharp et al. 1989). pSymB of *S. meliloti* has been known to house essential genes (Cooper et al. 2010; Morrow and Cooper 2012), and *Streptomyces* has majority of its essential genes concentrated near the origin of replication (Bentley et al. 2002; Kirby 2011). Additionally, pSymB has been shown to be more transcriptionally integrated with the chromosome compared with pSymA (DiCenzo et al. 2018), potentially contributing to the location of essential genes. Some of the proteins encoded on pSymB, which are not necessarily deemed essential, are still able to fulfill essential gene roles and functions (DiCenzo et al. 2018). These essential genes should have a decreased number of substitutions and therefore, coincide with the increasing substitution rate when moving away from the origin of replication in *Streptomyces* and pSymB of *S. meliloti*.

Molecular composition, gene content, and replication may all be factors contributing to the curious decreasing number of substitutions with increasing genomic distance found in *E. coli* and the chromosome of *S. meliloti* in this study. The integration of new genetic information through gene gain and loss sometimes occurs in particular regions along bacterial genomes termed “hotspots” (Streisinger et al. 1966; Farabaugh et al. 1978; Touchon et al. 2009; Oliveira et al. 2017). The frequency of these hotspots increases linearly with distance from the origin of replication (Oliveira et al. 2017), although different mobile elements, such as integrative and conjugative elements and prophages, appear to have a different distribution (Oliveira et al. 2017). Variation in these preferential sites for gene gain and loss could be located near the origin of replication and may illuminate why we observed the number of substitutions to significantly decrease with distance from the origin of replication in the chromosomes of *E. coli* and *S. meliloti*. Some studies found inconsistencies, with the placement of core genes concentrated near the terminus or distributed evenly throughout the genome, rather than localized at the origin of replication (Kopejtka et al. 2019). Determining the distribution and placement of the core and accessory genes in *E. coli*, and *S. meliloti* could elucidate why these replicons appear to have a higher number of substitutions near the origin of replication. The distinct placement of genes across the genome is speculated to be in part due to the nature of replication. Translocations can happen at replication forks as they advance along the chromosome (Tillier and Collins 2000; Mackiewicz et al. 2001). If these replication forks were concentrated near the origin of replication, creating a hotspot for an increased number of translocations present in that area, providing an opportunity for new genomic signatures to arise, such as a minor increase in the number of substitutions near the origin of replication.

Additionally, potential genomic and pathogenicity islands have been found near the origin of replication in *Mycobacterium tuberculosis* and *Haloquadratum walsbyi* (Karlin 2001; Mira et al. 2010). These islands were found to have genomic signatures such as codon bias, which deviated from the rest of the genome (Karlin 2001). Deviations in these genomic signatures may extend to substitution rates and provide another potential explanation as to why some of the replicons in this study had a slight increase in the number of substitutions near the origin of replication. Other genomic signatures such as GC content or nucleotide composition have been found to significantly change around the origin of replication and terminus (Mackiewicz et al. 1999; Ikeda et al. 2003), and may be a contributing factor in explaining a higher number of substitutions near the origin of replication in *E. coli* and the chromosome of *S. meliloti*, and warrants further investigation.

Rearrangements, inversions, duplications, and HGT all play a major role in shaping gene order, gene expression, gene content, and substitutions in bacterial replicons. One study found that the density of transposon insertion events peaks at the origin of replication and is at a minimum at the terminus in *E. coli* (Gerdes et al. 2003). Once again, the differences in various genomic signatures caused by genome reorganization, in this case transposon insertion events, may be a justification for the high number of substitutions seen near the origin in some chromosomes in this analysis. The lack of a clear spatial genomic substitution trend in the genomes used, highlights the importance of accounting for genomic reorganization, such as rearrangements, in molecular analysis.

### Spatial Selection Trends

Looking at the correlation between d*N*, d*S*, and *ω* values and distance from the origin of replication, we were unable to confirm a consistent linear correlation in the genomes analyzed (table 4 and figs. 5 and 6). There are a few sparse areas in the distribution of d*N*, d*S*, and *ω* values across the genomes. These are areas where alignment data were removed due to poor homology, excessive gaps, or not being present in all taxa. We manually looked into genes with unusually high values of d*N* and d*S*, and we have determined that these values indeed represent genes with a high number of substitutions. The substitutions in these genes often have many (or only) substitutions of one type (i.e., synonymous or nonsynonymous), skewing the d*N* or d*S* calculation, causing the unusually high values. These genes can be assumed to have a high degree of divergence between the taxa and often encode for unconfirmed proteins such as hypothetical proteins (see Supplementary Material online). Conversely, all *S. meliloti* chromosomes used in this analysis are extremely similar and therefore resulting in an overall low number of substitutions. The majority (61%) of the genes and gene segments in the chromosome of *S. meliloti* had d*N* values of 0, and therefore *ω* values of 0 (Supplementary Material online). These zero values were not removed from the analysis or outlier calculations because they were too numerous to be outliers and they provide important information about the similarities between these strains of *S. meliloti*. The low number of substitutions and consequently high numbers of zero d*N*, d*S*, and *ω* values in this bacteria are reflected in figure 6.

As mentioned previously, the number of bacterial genomes used for each analysis was limited partially due to computational constraints completing the progressiveMauve whole-genome alignment. Specialized alignment programs such as Parsnp (Treangen et al. 2014) identify and align only core regions of the genomes relatively quickly. Dealing with only core regions would reduce the potential for including alignments of poor sequence homology. This could allow the current analysis to be expanded to include more genomes of each bacterial species and potentially add more phylogenetic diversity in the species chosen. However, using only the core genome removes valuable data from the analysis such as accessory genes, where most variations in mutation rate would be seen (Couturier and Rocha 2006; Flynn et al. 2010).

This work is not the first to observe diverging results from the general consensus of bacterial molecular trends. These notable exceptions to what are thought to be generally applicable rules of bacterial molecular trends, question the broad universal assumption of these phenomenon. With respect to mutations, there was a number of studies that were unable to confirm a positive linear correlation between distance from the origin of replication and mutation rates (Hudson et al. 2002; Ochman 2003; Juurik et al. 2012; Martina et al. 2012; Dettman et al. 2016; Dillon et al. 2018). Some of these patterns are thought to be a regional effect of sequence composition (Hudson et al. 2002), whereas others are more related to cell cycle function (Dillon et al. 2018). There are a number of other intertwining factors that impact the mutation spectra of bacteria such as transcription, replication, and growth state (Hudson et al. 2002; Ochman 2003; Juurik et al. 2012). When looking at differences in mutations between replicons of the multirepliconic bacteria *Burkholderia*, substitutions are highest on the primary chromosomes compared with the secondary replicons (Dillon et al. 2015). This finding was unrelated to nucleotide composition and due to some substitutions occurring at higher rates on particular replicons (Dillon et al. 2015).

## Conclusions

The integration of genomic reorganization, such as rearrangements and inversions, can have impacts on spatial molecular trends such as substitution rate. The general molecular trends previously found in bacteria when moving away from the origin of replication may not be as commonplace as expected particularly when genome reorganization occurs. By utilizing ancestral reconstruction, we have demonstrated how information on genomic reorganization can be used to elucidate the spatial pattern of substitutions along bacterial genomes. We have illustrated that overarching spatial molecular trends may not be as universal as previously thought. We have found significant but small and inconsistent correlations between the number of substitutions and distance from the origin of replication in the genomes analyzed. We did not observe a consistent significant correlation between d*N*, d*S*, and *ω* values and distance from the origin of replication in the genomes analyzed. Combining genomic reorganization and current molecular pipelines through processes, such as ancestral reconstruction, can add vital information to bacterial genome analysis. We believe that genomic location and genome reorganization are important to consider in future molecular evolutionary analysis in all areas such as gene expression, essential gene locations, and functional classification of those genes. Observing other molecular trends through the lens of genomic reorganization will assist in answering questions about the evolution of bacteria.

## Supplementary Material

Supplementary data are available at *Genome Biology and Evolution* online.

## Supplementary Material

evaa260_Supplementary_Data

## Data Availability

The data underlying this article are available on GitHub at www.github.com/dlato/Location_of_Substitutions_and_Bacterial_Arrangements.
