# Supplementary material for: The Location of Substitutions and Bacterial Genome Arrangements
Source: Genome Biol Evol. 2020 Dec 15;13(1):evaa260. doi: 10.1093/gbe/evaa260 (PMC7851589; doi:10.1093/gbe/evaa260)
Supplement: evaa260_Supplementary_Data [file evaa260_supplementary_data.pdf]

Title: The Location of Substitutions and Bacterial Genome Arrangements

Authors: Daniella F. Lato and G. Brian Golding

Journal: Genome Biology and Evolution

Corresponding Author Information:
G. Brian Golding
McMaster University
Department of Biology
1280 Main St. West
Hamilton, ON
Canada
L8S 4K1
Email: golding@mcmaster.ca

Supplementary Material

For the most up to date Supplementary Material, please visit www.github.com/dlato/Location_of_Substitutions_and_Bacterial_Arrangements.

Further supplemental information and code are available on GitHub at www.github.com/dlato/Location_of_Substitutions_and_Bacterial_Arrangements.

Software Version Numbers

Program	Version Number	Build Date
baseml	4.9	March 2015
codeml	4.9	March 2015
consense	3.6b	NA
dnadist	3.6b	NA
dnaml	3.6b	NA
MAFFT	v7.045b	June 5, 2013
neighbor	3.6b	NA
progressiveMauve	Snap Shot	June 7, 2012
RAxML	8.0.25	June 16, 2014
seqboot	3.6b	NA
trimAl	v1.4.rev15	December 17, 2013

Table S1: Version numbers and build dates for each of the programs used.

Sequences

Bacteria Strain/Species	Accession Number	Date Accessed
Escherichia coli		
E. coli 0104H4	CP003289	September 29, 2016
E. coli 0157H7	BA000007	September 29, 2016
E. coli 083H1	CP001855	September 29, 2016
E. coli IAI39	CU928164	September 26, 2016
E. coli K12 *	U00096	September 26, 2016
E. coli UMN026	CU928163	September 26, 2016
Outgroup: *E. fergusonii* ATCC 35469T	NC_011740	August 26, 2020
Bacillus subtilis		
B. subtilis 168 *	NC_000964	November 10, 2016
B. subtilis BS38	NZ_CP017314	November 11, 2016
B. subtilis BSn5	NC_014976	November 11, 2016
B. subtilis PY79	NC_022898	November 11, 2016
B. subtilis QB928	NC_018520	November 11, 2016
B. subtilis RONN1	NC_017195	November 11, 2016
B. subtilis W23	NC_014479	November 11, 2016
Outgroup: *B. cereus* FDAARGOS_797	NZ_CP053931	August 26, 2020
Streptomyces		
S. lividans TK24	NZ_GG657756	August 26, 2020
S. lividans 1362	NZ_CM001889	August 26, 2020
S. coelicolor A3 *	AL645882	November 30, 2016
S. coelicolor A32 CFB NCB	NZ_CP042324	August 26, 2020
S. coelicolor M1154/pAMX4/pGP1416	NZ_CP050522	August 26, 2020
Outgroup: *S. aureofaciens* DM1	NZ_CP020567	August 26, 2020
S. meliloti Chromosome		
S. meliloti 2011	NC_020528	April 24, 2017
S. meliloti 1021 *	NC_003047	June 3, 2014
S. meliloti AK83	NC_015590	June 3, 2014
S. meliloti BL225C	NC_017322	June 3, 2014
S. meliloti SM11	NC_017325	June 3, 2014
S. meliloti RMO17	NC_CP009144	April 24, 2017
Outgroup: *Rhizobium leguminosarum* trifolii WSM1689 chromosome	NZ_CP007045	August 26, 2020
S. meliloti pSymA		
S. meliloti 2011	NC_020527	April 24, 2017
S. meliloti 1021 *	NC_003037	June 3, 2014
S. meliloti AK83	NC_015591	June 3, 2014
S. meliloti BL225C	NC_017324	June 3, 2014
S. meliloti SM11	NC_017327	June 3, 2014
S. meliloti RMO17	NC_CP009145	April 24, 2017
Outgroup: *Rhizobium leguminosarum* trifolii WSM1689 plasmid pRLG202	NC_0113665	August 26, 2020
S. meliloti pSymB		
S. meliloti 2011	NC_020560	April 24, 2017
S. meliloti 1021 *	NC_003078	June 3, 2014
S. meliloti AK83	NC_015596	June 3, 2014
S. meliloti BL225C	NC_017323	June 3, 2014
S. meliloti SM11	NC_017326	June 3, 2014
S. meliloti RMO17	NC_CP009146	April 24, 2017
Outgroup: *Rhizobium leguminosarum* trifolii WSM1689 plasmid pRLG201	NC_011368	August 26, 2020

Table S2: Strains and species used for each replicon analysis. Accession numbers, date accessed, and outgroups for each replicon are provided. An astrix (*) insicates the strain that was used as the representative strain.

Constraints to Number of Sequence Chosen

Computational time constraints and the nature of the data were limiting factors for the number of strains that were chosen for each bacterial species. `progressiveMauve` is a multiple sequence alignment program which is useful for accounting for local and large scale genomic rearrangements. Some of the bacterial strains are very similar and therefore there was no issue finding a sufficient number of locally co-linear blocks (LCBs) without having the genomes broken into an overwhelming number of blocks. We had to strike a balance between having as many genomes in the analysis as possible, and comparing correct homologous sequences. The more distantly related the taxa are, the resulting `progressiveMauve` alignment contained shorter LCBs and many blocks that compared sequences of poor homology. This can be seen in an example of six *Streptomyces* genomes resulting in the genome being split into 521 LCBs (Supplementary Figure S1). Consequently, we had to reduce the number of genomes used for this analysis and after many iterations of genome combinations, we settled on the genomes listed in Table S2. This allowed for the correct comparison of homologous sequences, while also accounting for recombination.

The computational time required to run `progressiveMauve` was an additional constraint that needed to be considered. `progressiveMauve` can align multiple whole genomes an identify regions that have been rearranged within the taxa provided. This process happens in relatively quick computational time, however, like most other programs, the addition of more data increased the amount of time required to complete the process. We ran multiple instances of `progressiveMauve` with varying numbers of *E. coli* genomes. These data points were connected using a locally estimated scatter plot smoothing method and confidence intervals. From this data, we determined that increasing the number of genomes exponentially increases the run time of `progressiveMauve`. It becomes impractical to align more than 27 genomes with `progressiveMauve`, as anything over that would take more than 24h to run. The estimated computational run time to complete the alignment of 100 genomes would take over a month. The total computational time additionally depends on the divergence of the sequences. 26 divergent *Streptomyces* genomes took just under a month to complete the `progressiveMauve` alignment. This information combined with `progressiveMauve`'s inability to pair homologous sequences in LCBs of distantly related taxa, has limited the total number of genomes we can use per taxa to a maximum of 7. This provides the most accurate data and the most reasonable analysis duration.

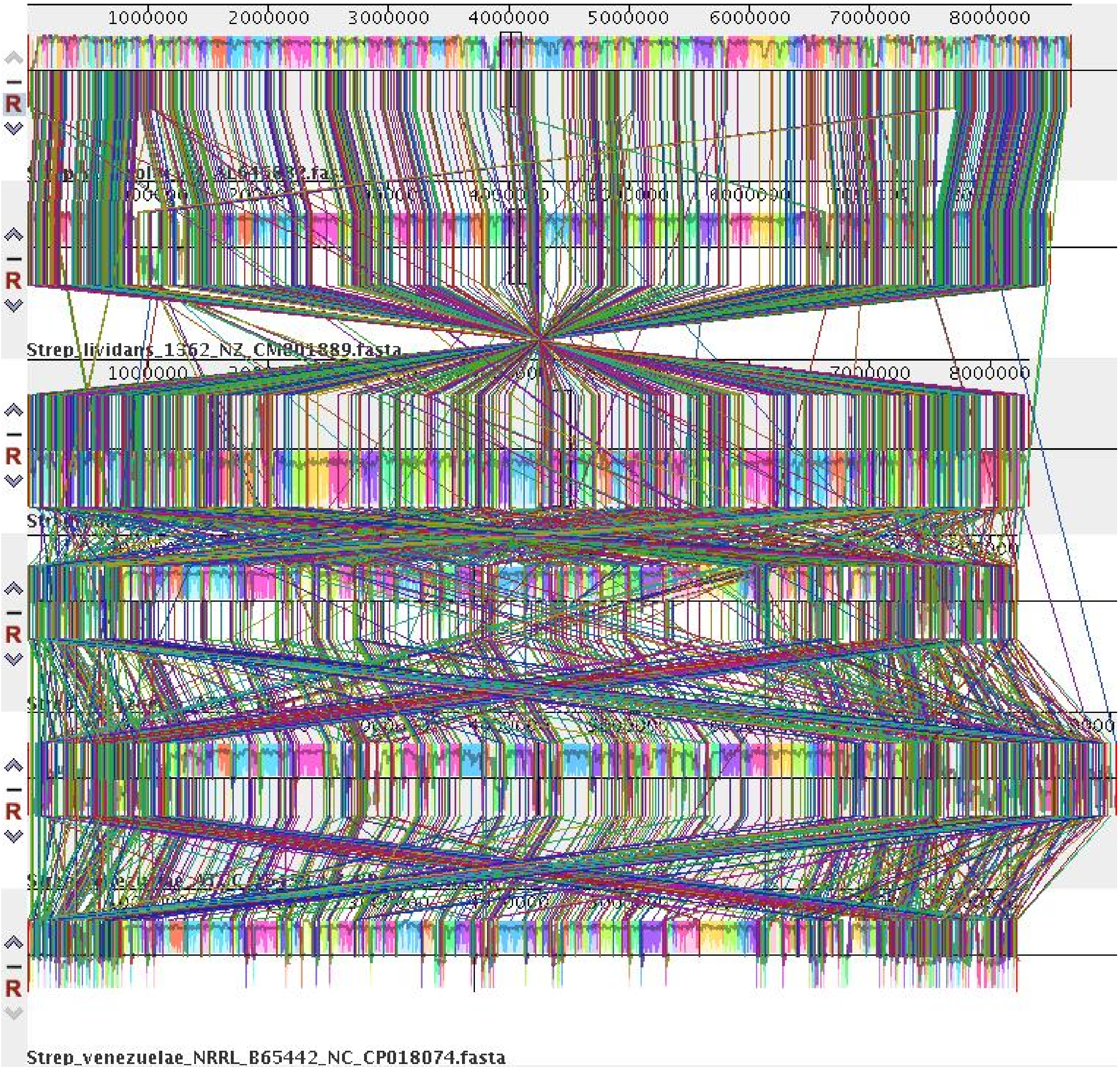

Figure S1: Visualization of the `progressiveMauve` alignment of 6 *Streptomyces* genomes (from top to bottom): *S. coelicolor* AL645882, *S. lividans* NZ_CM001889, *S. lividans* NZ_GG657756, *S. venezuelae* NC_018750, *S. venezuelae* NZ_CP013129, and *S. venezuelae* NC_CP018074. Each coloured block represents a different locally co-linear block (LCB). Coloured lines connect LCBs that are similar between taxa. The black lines underneath each LCB represent the whole genome sequence of each of the *Streptomyces* taxa. Each LCB can be treated as a rearrangement, there have therefore been 521 rearrangements between these *Streptomyces* genomes.

progressiveMauve Alignment

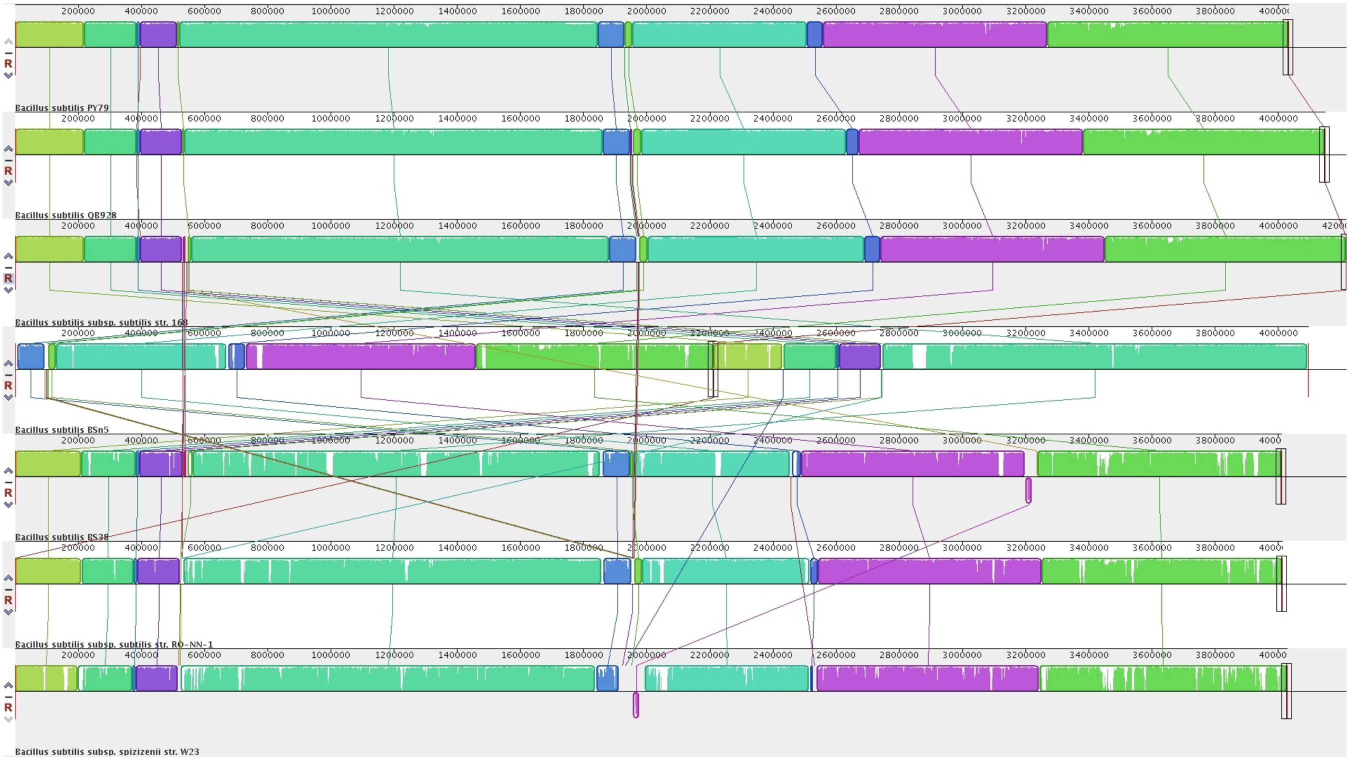

Figure S2: Visualization of the `progressiveMauve` alignment of the *B. subtilis* genomes. Each coloured block represents a different locally colinear block (LCB). Coloured lines connect LCBs that are similar between taxa. The black lines underneath each LCB represent the whole genome sequence of each of the *B. subtilis* taxa. From top to bottom the taxa are: *B. subtilis* PY79, *B. subtilis* QB928, *B. subtilis* 168, *B. subtilis* BSn5, *B. subtilis* BS38, *B. subtilis* RONN1, *B. subtilis* W23. Each LCB can be treated as a rearrangement, there have therefore been 12 rearrangements between these *B. subtilis* genomes.

Poor Sequence Alignment

After a re-alignment of `progressiveMauve` LCBs with `MAFFT` there were still regions of the alignment that were visibly poor. This prompted the additional alignment quality trimming using a custom `Python` script and `trimAl` (Capella-Gutiérrez et al. 2009). An example of what a "poor" alignment would look like can be found in Figure S3. The `FASTA` format of this segment of the alignment can be found on `GitHub` labelled as file "`poor_ecoli_alignment_example.fna`".

This segment of `MAFFT` alignment (Figure S3) appears to have completely misaligned the second sequence (*E. coli* O157H7). When we look at the genes that these regions of DNA are found within (Table S3), we see that the second sequence (*E. coli* O157H7) does not have the same protein sequence as the other bacteria genes. Poor sequence alignments like this, as well as other non-homologous alignment regions were removed from the analysis. Please see the main paper for more detailed methods.

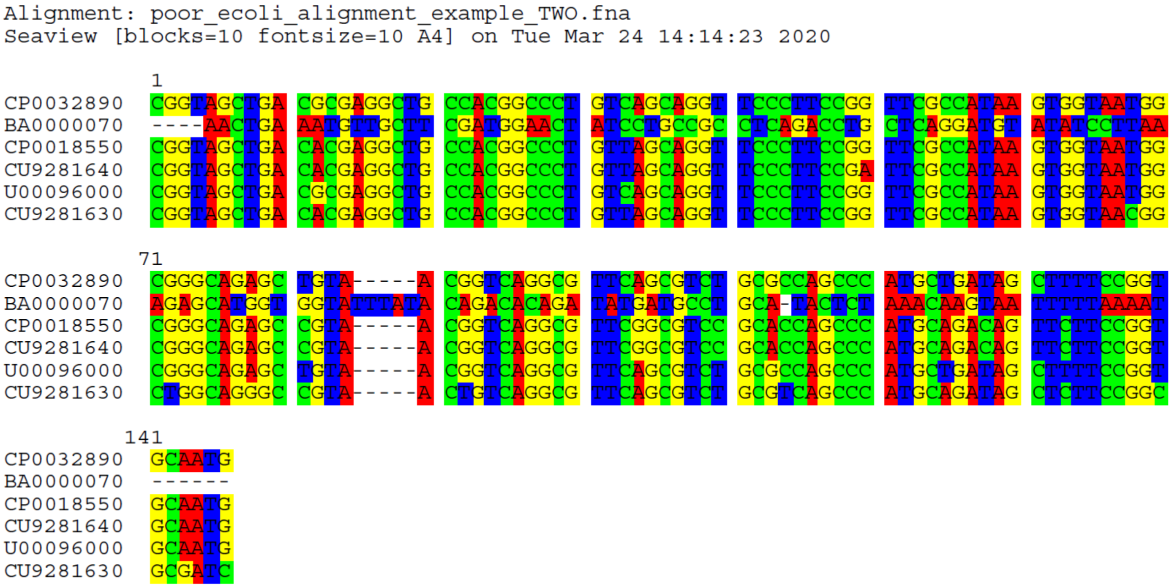

Figure S3: Visualization of a section of `MAFFT` alignment between the six strains of *E. coli*. This alignment was visualized with the `SeaView` graphical interface (Gouy et al. 2010).

E. coli Strain	NCBI Accession Number	Alignment Gene Id
0104H4	CP003289	O3K_04155
O157H7	BA000007	ECs3861
083H1	CP001855	NRG857_18350
IAI39	CU928164	yghE
K12	U00096	yghE
UMN026	CU928163	yghE

Table S3: *E. coli* strain, NCBI accession number, and Gene Id for the genes in the poor alignment example (Figure S3).

Phylogenetic Trees

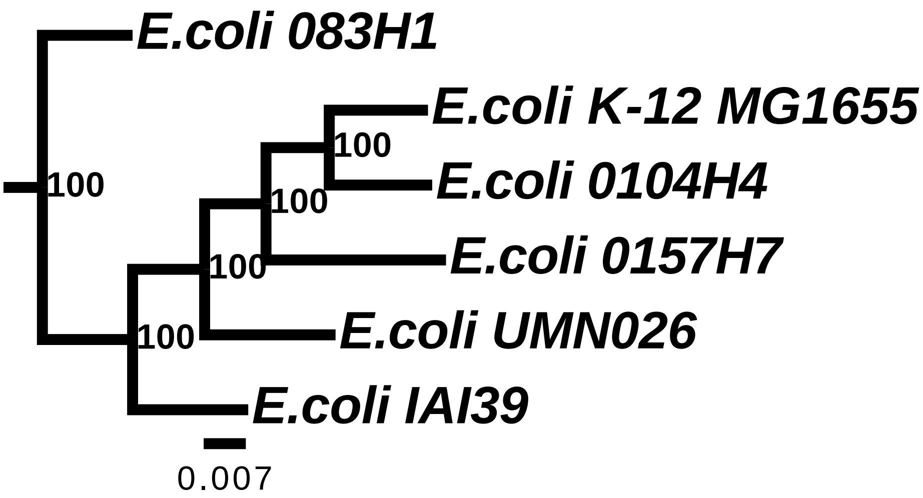

Figure S4: Phylogenetic tree of *E. coli* genomes. *E. fergusonii* ATCC 35469T was used as an outgroup to root the tree. Branch lengths are to scale. The numbers at each node indicate the bootstrap value as a percentage. The number of bootstrapped trees was 1000.

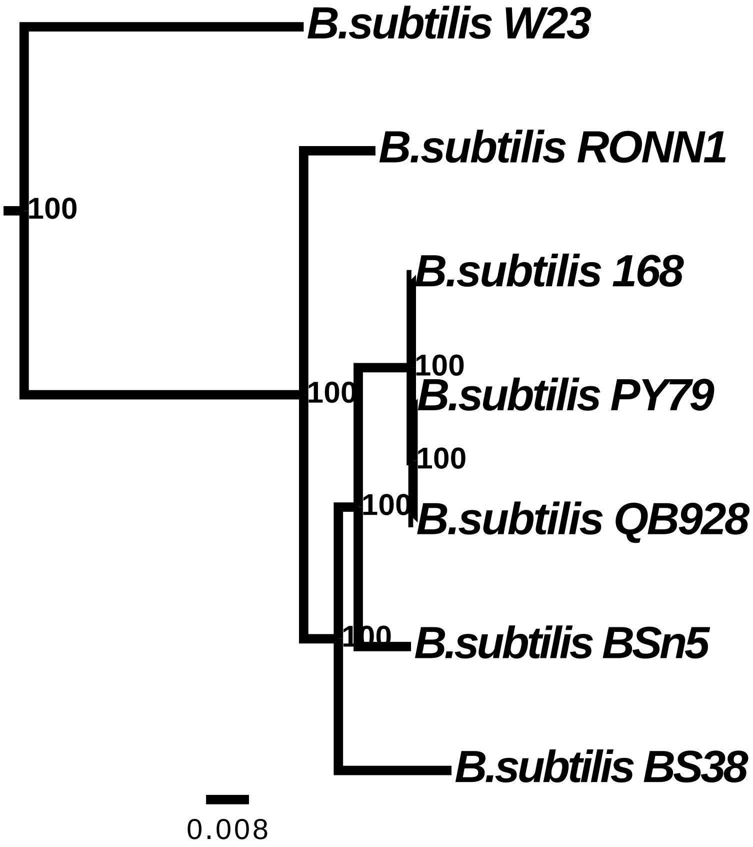

Figure S5: Phylogenetic tree of *B. subtilis* genomes. *B. cereus* FDAARGOS_797 was used as an outgroup to root the tree. Branch lengths are to scale. The numbers at each node indicate the bootstrap value as a percentage. The number of bootstrapped trees was 1000.

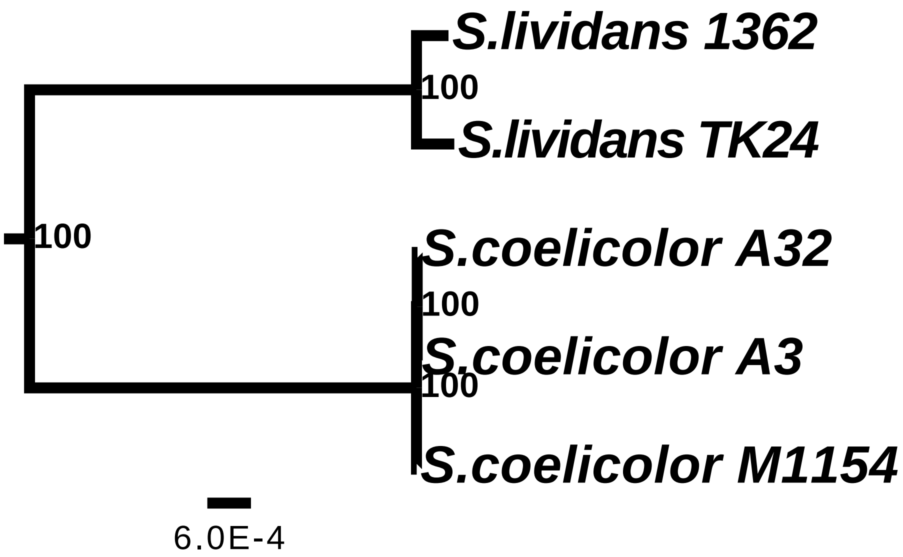

Figure S6: Phylogenetic tree of *Streptomyces* genomes. *S. aureofaciens* DM1 was used as an outgroup to root the tree. Branch lengths are to scale. The numbers at each node indicate the bootstrap value as a percentage. The number of bootstrapped trees was 1000.

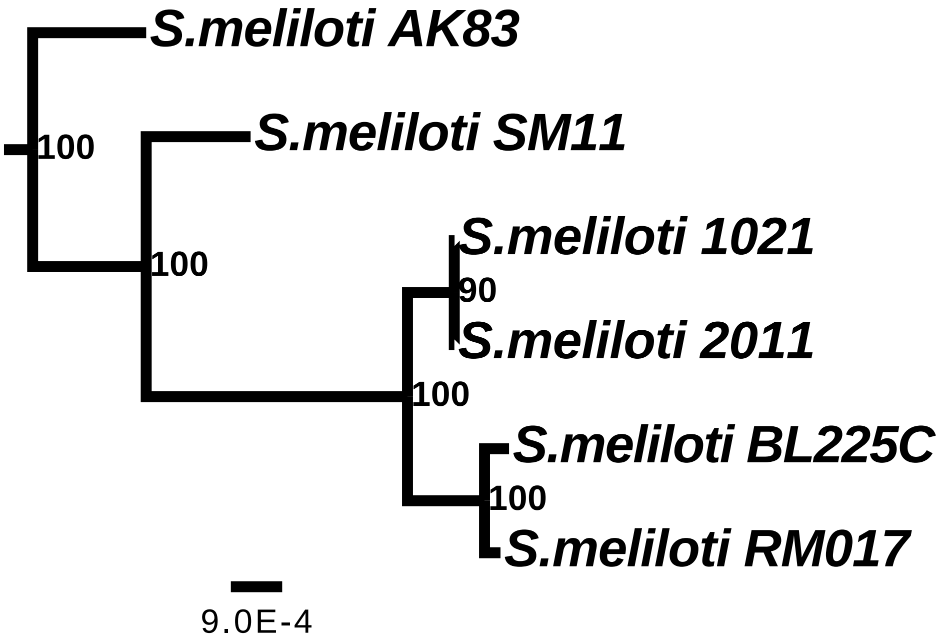

Figure S7: Phylogenetic tree using only the chromosomes of *S. meliloti*. *Rhizobium leguminosarum* trifolii WSM1689 chromosome was used as an outgroup to root the tree. Branch lengths are to scale. The numbers at each node indicate the bootstrap value as a percentage. The number of bootstrapped trees was 1000.

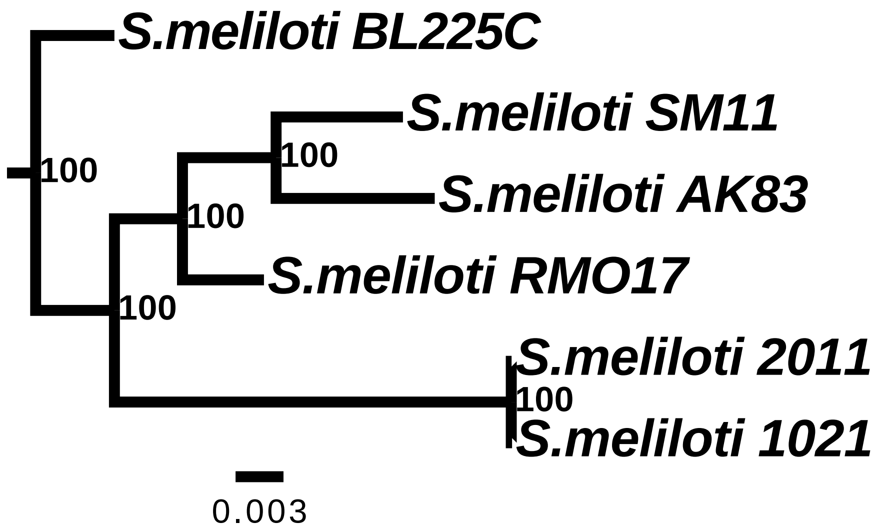

Figure S8: Phylogenetic tree using only pSymA of *S. meliloti*. *Rhizobium leguminosarum* trifolii WSM1689 plasmid pRLG202 was used as an outgroup to root the tree. Branch lengths are to scale. The numbers at each node indicate the bootstrap value as a percentage. The number of bootstrapped trees was 1000.

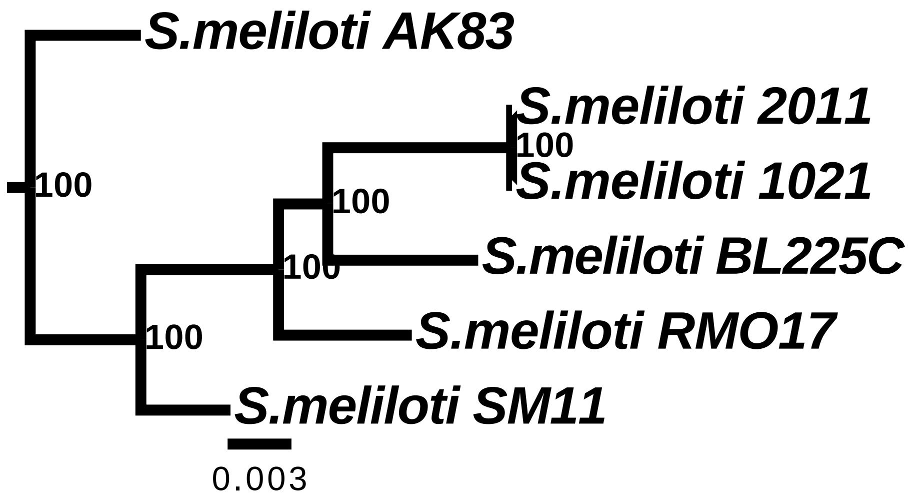

Figure S9: Phylogenetic tree using only pSymB of *S. meliloti*. *Rhizobium leguminosarum* trifolii WSM1689 plasmid pRLG201 was used as an outgroup to root the tree. Branch lengths are to scale. The numbers at each node indicate the bootstrap value as a percentage. The number of bootstrapped trees was 1000.

Origin and Terminus Locations

Each of the bacterial strains used in this analysis vary in total genomic length, in some cases this difference is up to 856Kbp like in *E. coli* (Table S4). This will cause the farthest point from the origin of replication to appear larger because of the increased genome size of some strains.

Bacteria	Origin of Replication	Terminus of Replication	Length of Longest Genome (bp)
E. coli	3925744	1588773	5498450
B. subtilis	1	1942542	4215606
Streptomyces	3419363	1 & 8667664	8667664
S. meliloti Chromosome	1	1735626	3908022
S. meliloti pSymA	1350001	672888	1633319
S. meliloti pSymB	55090	896756	1690594

Table S4: Origin of replication and terminus of replication positions in replicons of *E. coli*, *B. subtilis*, *Streptomyces*, and *S. meliloti*. The origin and terminus of replication are values from the representative strain of each bacteria, which can be found in Supplementary Table S2. The linear nature of *Streptomyces* chromosome gives it two termini, one at each end of the chromosome. The length of the longest genome is the longest genome length from all strains/species of each bacteria. This is not necessarily the same as the genome length of the representative strain.

Origin Location	*E. coli* Chromosome	*B. subtilis* Chromosome	*Streptomyces* Chromosome	*S. meliloti* Chromosome	*S. meliloti* pSymA	*S. meliloti* pSymB
Moved 100kb Left	$-1.445\times10^{-7***}$	$4.374\times10^{-9*}$	$6.909\times10^{-9***}$	$-1.316\times10^{-6***}$	$-1.058\times10^{-6***}$	$-2.009\times10^{-7***}$
Moved 90kb Left	$-1.544\times10^{-7***}$	$-1.036\times10^{-7***}$	$5.677\times10^{-9***}$	$-1.32\times10^{-6***}$	$-1.246\times10^{-6***}$	$-1.357\times10^{-7***}$
Moved 80kb Left	$-1.65\times10^{-7***}$	$-1.072\times10^{-7***}$	$8.11\times10^{-9***}$	$-1.338\times10^{-6***}$	$-1.398\times10^{-6***}$	$-6.57\times10^{-8***}$
Moved 70kb Left	$-1.667\times10^{-7***}$	$-1.102\times10^{-7***}$	$6.716\times10^{-9***}$	$-1.363\times10^{-6***}$	$-1.405\times10^{-6***}$	9.83×10^{-8}
Moved 60kb Left	$-1.64\times10^{-7***}$	$-1.19\times10^{-7***}$	$8.7\times10^{-9***}$	$-1.324\times10^{-6***}$	$-1.394\times10^{-6***}$	$1.129\times10^{-7***}$
Moved 50kb Left	$-1.446\times10^{-7***}$	$-1.211\times10^{-7***}$	$1.045\times10^{-8***}$	$-1.36\times10^{-6***}$	$-1.403\times10^{-6***}$	$1.521\times10^{-7***}$
Moved 40kb Left	$-1.4\times10^{-7***}$	$-1.299\times10^{-7***}$	$1.214\times10^{-8***}$	$-1.255\times10^{-6***}$	$-1.422\times10^{-6***}$	$1.543\times10^{-7***}$
Moved 30kb Left	$-1.498\times10^{-7***}$	$-1.292\times10^{-7***}$	$1.24\times10^{-8***}$	$-1.26\times10^{-6***}$	$-1.392\times10^{-6***}$	$1.63\times10^{-7***}$
Moved 20kb Left	$-1.51\times10^{-7***}$	$-1.1\times10^{-7***}$	$1.395\times10^{-8***}$	$-1.525\times10^{-6***}$	$-1.412\times10^{-6***}$	$1.603\times10^{-7***}$
Moved 10kb Left	$-1.262\times10^{-7***}$	-2.602×10^{-9}	$1.563\times10^{-8***}$	$-1.599\times10^{-6***}$	$-9.499\times10^{-7***}$	$2.973\times10^{-7***}$
Moved 10kb Right	$-1.305\times10^{-7***}$	$-2.045\times10^{-8***}$	$1.578\times10^{-8***}$	$1.614\times10^{-6***}$	$-1.026\times10^{-6***}$	$3.505\times10^{-7***}$
Moved 20kb Right	$-1.454\times10^{-7***}$	$-1.006\times10^{-7***}$	$1.903\times10^{-8***}$	$-1.634\times10^{-6***}$	$-1.475\times10^{-6***}$	$1.649\times10^{-7***}$
Moved 30kb Right	$-1.548\times10^{-7***}$	$-8.596\times10^{-8***}$	$2.046\times10^{-8***}$	$-1.698\times10^{-6***}$	$-1.417\times10^{-6***}$	$1.526\times10^{-7***}$
Moved 40kb Right	$-1.632\times10^{-7***}$	$-8.378\times10^{-8***}$	$2.125\times10^{-8***}$	$-1.719\times10^{-6***}$	$-1.367\times10^{-6***}$	$1.589\times10^{-7***}$
Moved 50kb Right	$-1.856\times10^{-7***}$	$-7.879\times10^{-8***}$	$1.957\times10^{-8***}$	$-1.735\times10^{-6***}$	$-1.277\times10^{-6***}$	$1.654\times10^{-7***}$
Moved 60kb Right	$-1.91\times10^{-7***}$	$-6.98\times10^{-8***}$	$1.974\times10^{-8***}$	$-1.788\times10^{-6***}$	$-1.169\times10^{-6***}$	$1.645\times10^{-7***}$
Moved 70kb Right	$-1.892\times10^{-7***}$	$-6.634\times10^{-8***}$	$1.934\times10^{-8***}$	$-1.854\times10^{-6***}$	$-1.059\times10^{-6***}$	$1.843\times10^{-7***}$
Moved 80kb Right	$-1.879\times10^{-7**}$	$-5.814\times10^{-8***}$	$2.313\times10^{-8***}$	$-1.891\times10^{-6***}$	$-9.07\times10^{-7***}$	$1.90\times10^{-7***}$
Moved 90kb Right	$-1.862\times10^{-7***}$	$-4.314\times10^{-8***}$	$2.304\times10^{-8***}$	$-1.865\times10^{-6***}$	$-7.171\times10^{-7***}$	$2.415\times10^{-7***}$
Moved 100kb Right	$-1.799\times10^{-7***}$	$-2.597\times10^{-8***}$	$1.945\times10^{-8***}$	$-1.525\times10^{-6***}$	$-6.572\times10^{-7***}$	$3.095\times10^{-7***}$

Table S5: Logistic regression analysis of the number of substitutions along the genome of the respective bacterial replicons after the origin location was moved by the specified increments from the original origin of replication position (listed in Table S4). All results are marked with significance codes as followed: < 0.001 = '***', $0.001 < 0.01$ = '**', $0.01 < 0.05$ = '*', $0.05 < 0.1$ = '.', > 0.1 = ' '. Logistic regression was calculated after the origin of replication was moved to the new location in the genome and all subsequent positions were scaled around the origin accounting for bidirectional replication.

Bacteria Strain	Accession Number	Date Accessed
E. coli K12 Chromosome	U00096	September 26, 2016
B. subtilis 168 Chromosome	NC_000964	November 10, 2016
S. coelicolor A3 Chromosome	AL645882	November 30, 2016
S. meliloti Chromosome 1021	NC_003047	June 3, 2014
S. meliloti pSymA 1021	NC_003037	June 3, 2014
S. meliloti pSymB 1021	NC_003078	June 3, 2014

Table S6: Strains and species used for determining the protein coding regions of each bacterial replicon. GenBank reference annotation was used to determine all protein coding sections of the replicons. NCBI accession numbers and date accessed are provided.

Genomic Position Clustering

A custom R script was used to cluster genomic positions together based on a user specified genetic distance using single-link clustering. An illustration of the clustering method used in this supplemental test can be found in Figure S10. This clustering was done for genomic distances beginning at 1bp and increasing by one order of magnitude until 1,000,000bp difference exists between the taxa genomic positions. These newly clustered genomic positions were then put into the same substitution analysis as mentioned previously to determine the impact of this position clustering on the spatial substitution trends through a linear regression. A complete table of the statistical results from the clustering assessment are found in Table S7. The results from this analysis indicate that genomic positions up to 1,000,000bp apart can be considered a singular genomic position without altering the overall spatial substitution analysis.

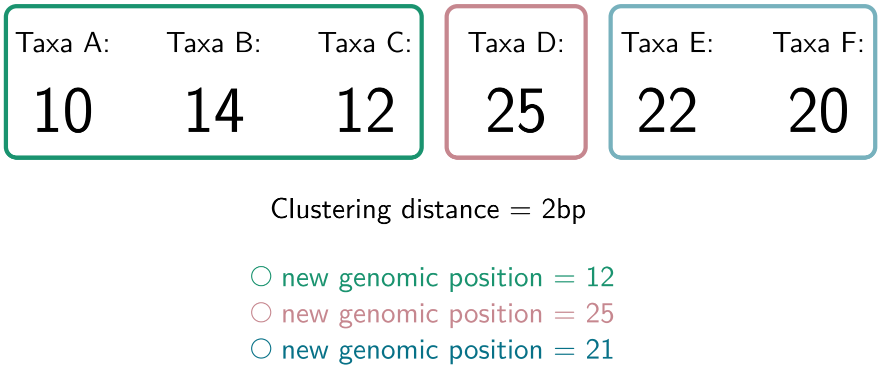

Figure S10: Visualization of the genomic position clustering method. In this example, the user specified the genetic distance to be 2, all genomic positions within 2 base pairs would be clustered together. In this example we are looking at 6 taxa with genomic positions 10, 14, 12, 25, 22, and 20. Based on the clustering algorithm, positions 10, 14 and 12 would be grouped into a cluster (outlined in green), position 25 would be its own cluster (outlined in pink), and positions 22 and 20 would be grouped into another cluster (outlined in blue). Once the clusters are determined, a new genomic position for each of the clusters is calculated using the average of all positions within that cluster. In this example, the green cluster would have a new genomic position of 12 (the average between those three positions), the pink cluster would have the sane genomic position of 25, and the blue cluster would have a new genomic position of 21. The new list of genomic positions for the 4 taxa would be: 12, 12, 12, 25, 21 and 21.

Position Difference	*E. coli* Chromosome	*B. subtilis* Chromosome	*Streptomyces* Chromosome	*S. meliloti* Chromosome	*S. meliloti* pSymA	*S. meliloti* pSymB
1bp	-1.394×10^{-7}**	-2.538×10^{-8}**	1.736×10^{-8}**	-1.541×10^{-6}**	-9.130×10^{-7}**	2.488×10^{-7}***
10bp	-1.394×10^{-7}***	-2.518×10^{-8}***	-4.484×10^{-9}***	-1.627×10^{-6}***	-9.13×10^{-7}***	3.487×10^{-7}***
100bp	-1.764×10^{-7}***	-1.417×10^{-8}***	1.448×10^{-8}***	-1.605×10^{-6}***	-1.166×10^{-6}***	4.021×10^{-7}***
1000bp	-1.784×10^{-7}***	-1.417×10^{-8}***	1.505×10^{-8}***	-1.605×10^{-6}***	-1.153×10^{-6}***	4.021×10^{-7}***
10000bp	-1.712×10^{-7}***	-3.496×10^{-8}***	4.790×10^{-8}***	-1.605×10^{-6}***	-3.570×10^{-8}*	3.784×10^{-7}***
100000bp	-2.061×10^{-7}***	-3.561×10^{-8}***	4.167×10^{-9}***	-1.605×10^{-6}***	-4.676×10^{-7}***	3.784×10^{-7}***
1000000bp	4.229×10^{-8}***	-7.710×10^{-9}***	6.083×10^{-8}***	-1.605×10^{-6}***	4.285×10^{-6}***	-8.888×10^{-7}***

Table S7: Results from the position clustering analysis. Logistic regression analysis of the number of substitutions along the genome of the respective bacteria replicons to test position differences. The “Position Difference” column denotes different base pair distances that the positions in the genome were clustered together as. All results are marked with significance codes as followed: < 0.001 = ‘***’, $0.001 < 0.01$ = ‘**’, $0.01 < 0.05$ = ‘*’, $0.05 < 0.1$ = ‘.’, > 0.1 = ‘ ’. Logistic regression was calculated after the positions in the genome were determined to be the same at each position difference listed in the first column.

Bacteria and Replicon	Average Replicon Length	Number of Sites	Number of Substitutions
E. coli Chromosome	5082529	3032961	200477
B. subtilis Chromosome	4077077	2411673	218843
Streptomyces Chromosome	8494093	5266854	20929
S. meliloti Chromosome	3426881	2125845	6420
S. meliloti pSymA	1455940	451314	10055
S. meliloti pSymB	1664597	1200129	28233

Table S8: Total number of protein coding sites in each replicon for this analysis and the number of those sites that have a substitution (multiple substitutions at one site are counted as two substitutions).

High Substitutions Gene Example

Throughout this analysis there are a few genes/gene segments in all the bacterial replicons that have relatively high numbers of substitutions when compared to other genes or gene segments. These high numbers of substitutions are indeed real changes seen in homologous genes. To illustrate this, we have chosen a segment of alignment from *B. subtilis*. Information about the genes involved in this segment can be found in Table S9. A protein alignment for these genes can be found on `GitHub` (`www.github.com/dlato/Location_of_Substitutions_and_Bacterial_Arrangements`) under the file name “`Bacillus_high_substitutions_gene_example.txt`”.

Despite this high sequence identity and almost identical protein alignment (Figures S11 and S12), there are a total of 205 substitutions (across all nodes of the phylogenetic tree, Figure S5) within this short stretch of sequence. It is segments like these that are resulting in the appearance of extremely high numbers of substitutions in sections of all the bacterial replicon genomes.

Species	NCBI Accession Number	Gene Id
B. subtilis 168	NC_000964	BSU17380
B. subtilis BS38	NZ_CP017314	BSBS38_RS09695
B. subtilis BSn5	NC_014976	BSN5_RS21150
B. subtilis PY79	NC_022898	U712_RS08990
B. subtilis QB928	NC_018520	B657_RS09460
B. subtilis RONN1	NC_017195	I33_RS09040
B. subtilis W23	NC_014479	BSUW23_RS09220

Table S9: Information about the example gene segment from *B. subtilis* alignment with high number of substitutions.

```
Alignment: Block21_coding_gene_aln_0852_sec_0001_good_sec_0000.txt
Seaview [blocks=10 fontsize=10 A4] on Tue Sep 29 13:39:03 2020

                           1
NC_000964:1869011-1871071  GAAATCATGA TCCAAAAAGA TGGGAAATTC CAGTTTGATA AGGATAAAGA
NC_CP01731:1840890-1842950 GAAATCATGA TCCAAAAAGA TGGAAAATTC CAGTTTGATA AGGATAAAGA
NC_014976:4079508-4081568  GAAATCATGA TCCAAAAAGA TGGAAAATTC CAGTTTGATA AGGATAAAGA
NC_022898:1832049-1834109  GAAATCATGA TCCAAAAAGA TGGGAAATTC CAGTTTGATA AGGATAAAGA
NC_018520:1848510-1850570  GAAATCATGA TCCAAAAAGA TGGGAAATTC CAGTTTGATA AGGATAAAGA
NC_017195:1843504-1845564  GAAATCATGA TCCAAAAAGA TGGAAAATTC CAGTTTGATA AGGATAAAGA
NC_014479:1826454-1828514  GAAATCATGA TCCAAAAAGA TGGAAAATTC CAGTTTGATA AGGATAAAGA

                          51
NC_000964:1869011-1871071  AGCTGTACAT AGCTATTTTG TAGATTATAT CAATCAAAAC ACAGTCTTCT
NC_CP01731:1840890-1842950 GGCTGTACAT AGCTATTTTG TAGATTATAT CAATCAAAAC ACAGTCTTCT
NC_014976:4079508-4081568  GGCTGTACAT AGCTATTTTG TAGATTATAT CAATCAAAAC ACAGTCTTCT
NC_022898:1832049-1834109  AGCTGTACAT AGCTATTTTG TAGATTATAT CAATCAAAAC ACAGTCTTCT
NC_018520:1848510-1850570  AGCTGTACAT AGCTATTTTG TAGATTATAT CAATCAAAAC ACAGTCTTCT
NC_017195:1843504-1845564  GGCTGTACAT AGCTATTTTG TAGACTATAT CAATCAAAAC ACAGTGTACT
NC_014479:1826454-1828514  GGCTGTACAT AGCTATTTTG TAGATTATAT CAATCAAAAC ACAGTTTTCT

                         101
NC_000964:1869011-1871071  TTCACAATTT AAAAGAGAAG CTGGATTATT TGGTTGAAAA CCAATACTAC
NC_CP01731:1840890-1842950 TTCACGATTT AAAAGAGAAG CTGGATTATT TGGTTGAAAA TCAATACTAC
NC_014976:4079508-4081568  TTCACGATTT AAAAGAGAAG CTGGATTATT TGGTTGAAAA CCAATACTAC
NC_022898:1832049-1834109  TTCACAATTT AAAAGAGAAG CTGGATTATT TGGTTGAAAA CCAATACTAC
NC_018520:1848510-1850570  TTCACAATTT AAAAGAGAAG CTGGATTATT TGGTTGAAAA CCAATACTAC
NC_017195:1843504-1845564  TTCACGATTT AAAAGAGAAG CTGGATTATT TGATTGAAAA TCAATACTAC
NC_014479:1826454-1828514  TTCACGACTT AAAAGAGAAG CTGGATTATT TGATTGAAAA CCAATACTAC

                         151
NC_000964:1869011-1871071  GAAGAGGAAT TCTTAAGCCT TTATTCTTTT GAAGACATTA AAGAAGTGTT
NC_CP01731:1840890-1842950 GAAGAGGAAT TCTTAAGCCT TTATTCTTTT GAAGACATTA AAGAAGTGTT
NC_014976:4079508-4081568  GAAGAGGAAT TCTTAAGCCT TTATTCTTTT GAAGACATTA AAGAAGTGTT
NC_022898:1832049-1834109  GAAGAGGAAT TCTTAAGCCT TTATTCTTTT GAAGACATTA AAGAAGTGTT
NC_018520:1848510-1850570  GAAGAGGAAT TCTTAAGCCT TTATTCTTTT GAAGACATTA AAGAAGTGTT
NC_017195:1843504-1845564  GAAGAGGAGT TCTTAAGCCT TTATTCTTTT GAAGACATTA AAGAAGTGTT
NC_014479:1826454-1828514  GAAGAGGAAT TCTTAAGCCT TTATTCTTTT GAAGACATTA AAGCAGTGTT

                         201
NC_000964:1869011-1871071  TAAGACAGCT TACGCTAAGA AGTTTCGTTT TCCTTCCTTC ATGAGTGCGT
NC_CP01731:1840890-1842950 TAAGACAGCT TACGCTAAGA AGTTTCGTTT TCCTTCCTTC ATGAGTGCGT
NC_014976:4079508-4081568  TAAGACAGCT TACGCTAAGA AGTTTCGTTT TCCTTCCTTC ATGAGTGCGT
NC_022898:1832049-1834109  TAAGACAGCT TACGCTAAGA AGTTTCGTTT TCCTTCCTTC ATGAGTGCGT
NC_018520:1848510-1850570  TAAGACAGCT TACGCTAAGA AGTTTCGTTT TCCTTCCTTC ATGAGTGCGT
NC_017195:1843504-1845564  TAAGACAGCT TACGCAAAGA AGTTTCGTTT TCCTTCCTTC ATGAGTGCGT
NC_014479:1826454-1828514  TAAGACAGCT TACGCTAAGA AGTTTCGTTT TCCTTCCTTC ATGAGTGCGT

                         251
NC_000964:1869011-1871071  TTAAATTCTA TAATGACTAT GCTTTGAAAA CGAATGACAA GAAAAAAATC
NC_CP01731:1840890-1842950 TTAAATTCTA TAATGACTAT GCTTTGAAAA CGAATGACAA GAAAAAAATC
NC_014976:4079508-4081568  TTAAATTCTA TAATGACTAT GCTTTGAAAA CGAATGACAA GAAAAAAATC
NC_022898:1832049-1834109  TTAAATTCTA TAATGACTAT GCTTTGAAAA CGAATGACAA GAAAAAAATC
NC_018520:1848510-1850570  TTAAATTCTA TAATGACTAT GCTTTGAAAA CGAATGACAA GAAAAAAATC
NC_017195:1843504-1845564  TTAAATTTTA TAATGACTAT GCTTTGAAAA CGAATGACAA GAAAAGAATC
NC_014479:1826454-1828514  TTAAATTCTA TAATGACTAT GCTTTGAAAA CGAATGACAA GAAAAAAATC

                         301
NC_000964:1869011-1871071  CTCGAGCGTT ATGAGGACCG GATCTCAATT GTTGCGCTGT TCTTCGCTAA
NC_CP01731:1840890-1842950 CTCGAGCGTT ATGAGGACCG GATCTCAATT GTTGCGCTGT TCTTCGCTAA
NC_014976:4079508-4081568  CTCGAGCGTT ATGAGGACCG GATCTCAATT GTTGCGCTGT TCTTCGCTAA
NC_022898:1832049-1834109  CTCGAGCGTT ATGAGGACCG GATCTCAATT GTTGCGCTGT TCTTCGCTAA
NC_018520:1848510-1850570  CTCGAGCGTT ATGAGGACCG GATCTCAATT GTTGCGCTGT TCTTCGCTAA
NC_017195:1843504-1845564  CTCGAGCGTT ATGAGGACCG GATCTCAATT GTTGCGCTGT TCTTCGCAAA
NC_014479:1826454-1828514  CTCGAGCGAT ATGAGGACCG AATCTCAATT GTCGCGCTTT TCTTCGCAAA

                         351
NC_000964:1869011-1871071  CGGCGACACT GAGAAAGCAA AAGAATATGT AAACCTGATG ATCAATCAAG
NC_CP01731:1840890-1842950 CGGCGACACT GAGAAAGCAA AAGAATATGT AAACCTGATG ATCAATCAAG
NC_014976:4079508-4081568  CGGCGACACT GAGAAAGCAA AAGAATATGT AAACCTGATG ATCAATCAAG
NC_022898:1832049-1834109  CGGCGACACT GAGAAAGCAA AAGAATATGT AAACCTGATG ATCAATCAAG
NC_018520:1848510-1850570  CGGCGACACT GAGAAAGCAA AAGAATATGT AAACCTGATG ATCAATCAAG
NC_017195:1843504-1845564  CGGCGACACT GAGAAAGCAA AGGAATATGT GCACCTGATG ATCAATCAGG
NC_014479:1826454-1828514  CGGCGACACT GAGAAAGCAA AAGAATATGT GAACCTGATG ATCAATCAGG
```

Figure S11: Visualization of a portion of the nucleotide alignment of *B. subtilis* genes with high numbers of substitutions. Alignment visualization was performed with `SeaView` (Gouy et al. 2010)

```
Alignment: Block21_coding_gene_aln_0852_sec_0001_good_sec_0000.txt
Seaview [blocks=10 fontsize=10 A4] on Tue Sep 29 13:50:41 2020

                            1
NC_000964:1869011-1871071   EIMIQKDGKF QFDKDKEAVH SYFVDYINQN TVFFHNLKEK LDYLVENQYY
NC_CP01731:1840890-1842950  EIMIQKDGKF QFDKDKEAVH SYFVDYINQN TVFFHDLKEK LDYLVENQYY
NC_014976:4079508-4081568   EIMIQKDGKF QFDKDKEAVH SYFVDYINQN TVFFHDLKEK LDYLVENQYY
NC_022898:1832049-1834109   EIMIQKDGKF QFDKDKEAVH SYFVDYINQN TVFFHNLKEK LDYLVENQYY
NC_018520:1848510-1850570   EIMIQKDGKF QFDKDKEAVH SYFVDYINQN TVFFHNLKEK LDYLVENQYY
NC_017195:1843504-1845564   EIMIQKDGKF QFDKDKEAVH SYFVDYINQN TVYFHDLKEK LDYLIENQYY
NC_014479:1826454-1828514   EIMIQKDGKF QFDKDKEAVH SYFVDYINQN TVFFHDLKEK LDYLIENQYY

                           51
NC_000964:1869011-1871071   EEEFLSLYSF EDIKEVFKTA YAKKFRFPSF MSAFKFYNDY ALKTNDKKKI
NC_CP01731:1840890-1842950  EEEFLSLYSF EDIKEVFKTA YAKKFRFPSF MSAFKFYNDY ALKTNDKKKI
NC_014976:4079508-4081568   EEEFLSLYSF EDIKEVFKTA YAKKFRFPSF MSAFKFYNDY ALKTNDKKKI
NC_022898:1832049-1834109   EEEFLSLYSF EDIKEVFKTA YAKKFRFPSF MSAFKFYNDY ALKTNDKKKI
NC_018520:1848510-1850570   EEEFLSLYSF EDIKEVFKTA YAKKFRFPSF MSAFKFYNDY ALKTNDKKKI
NC_017195:1843504-1845564   EEEFLSLYSF EDIKEVFKTA YAKKFRFPSF MSAFKFYNDY ALKTNDKKRI
NC_014479:1826454-1828514   EEEFLSLYSF EDIKAVFKTA YAKKFRFPSF MSAFKFYNDY ALKTNDKKKI

                          101
NC_000964:1869011-1871071   LERYEDRISI VALFFANGDT EKAKEYVNLM INQEYQPSTP TFLNAGRKRR
NC_CP01731:1840890-1842950  LERYEDRISI VALFFANGDT EKAKEYVNLM INQEYQPSTP TFLNAGRKRR
NC_014976:4079508-4081568   LERYEDRISI VALFFANGDT EKAKEYVNLM INQEYQPSTP TFLNAGRKRR
NC_022898:1832049-1834109   LERYEDRISI VALFFANGDT EKAKEYVNLM INQEYQPSTP TFLNAGRKRR
NC_018520:1848510-1850570   LERYEDRISI VALFFANGDT EKAKEYVNLM INQEYQPSTP TFLNAGRKRR
NC_017195:1843504-1845564   LERYEDRISI VALFFANGDT EKAKEYVHLM INQEYQPSTP TFLNAGRKRR
NC_014479:1826454-1828514   LERYEDRISI VALFFANGDT EKAKEYVNLM INQEYQPSTP TFLNAGRKRR

                          151
NC_000964:1869011-1871071   GELVSCFLLE VNDSLNDISR AIDISMQLSK LGGGVSLNLS KLRAKGEAIK
NC_CP01731:1840890-1842950  GELVSCFLLE VNDSLNDISR AIDISMQLSK LGGGVSLNLS KLRAKGEAIK
NC_014976:4079508-4081568   GELVSCFLLE VNDSLNDISR AIDISMQLSK LGGGVSLNLS KLRAKGEAIK
NC_022898:1832049-1834109   GELVSCFLLE VNDSLNDISR AIDISMQLSK LGGGVSLNLS KLRAKGEAIK
NC_018520:1848510-1850570   GELVSCFLLE VNDSLNDISR AIDISMQLSK LGGGVSLNLS KLRAKGEAIK
NC_017195:1843504-1845564   GELVSCFLLE VNDSLNDISR AIDISMQLSK LGGGVSLNLS KLRAKGEAIK
NC_014479:1826454-1828514   GELVSCFLLE VNDSLNDISR AIDISMQLSK LGGGVSLNLS KLRAKGEAIK

                          201
NC_000964:1869011-1871071   DVENATKGVV GVMKLLDNAF RYADQMGQRQ GSGAAYLNIF HRDINDFLDT
NC_CP01731:1840890-1842950  DVENATKGVV GVMKLLDNAF RYADQMGQRQ GSGAAYLNIF HRDINDFLDT
NC_014976:4079508-4081568   DVENATKGVV GVMKLLDNAF RYADQMGQRQ GSGAAYLNIF HRDINDFLDT
NC_022898:1832049-1834109   DVENATKGVV GVMKLLDNAF RYADQMGQRQ GSGAAYLNIF HRDINDFLDT
NC_018520:1848510-1850570   DVENATKGVV GVMKLLDNAF RYADQMGQRQ GSGAAYLNIF HRDINDFLDT
NC_017195:1843504-1845564   DVENATKGVV GVMKLLDNAF RYADQMGQRQ GSGAAYLNIF HRDINDFLDT
NC_014479:1826454-1828514   DVENATKGVV GVMKLLDNAF RYADQMGQRQ GSGAAYLNIF HRDINDFLDT

                          251
NC_000964:1869011-1871071   KKISADEDVR VKTLSIGVVI PDKFVELARE DKAAYVFYPH TIYKEYGQHM
NC_CP01731:1840890-1842950  KKISADEDVR VKTLSIGVVI PDKFVELARE DKAAYVFYPH TIYKEYGQHM
NC_014976:4079508-4081568   KKISADEDVR VKTLSIGVVI PDKFVELARE DKAAYVFYPH TIYKEYGQHM
NC_022898:1832049-1834109   KKISADEDVR VKTLSIGVVI PDKFVELARE DKAAYVFYPH TIYKEYGQHM
NC_018520:1848510-1850570   KKISADEDVR VKTLSIGVVI PDKFVELARE DKAAYVFYPH TIYKEYGQHM
NC_017195:1843504-1845564   KKISADEDVR VKTLSIGVVI PDKFVELARE DKAAYVFYPH TIYKEYGQHM
NC_014479:1826454-1828514   KKISADEDVR VKTLSIGVVI PDKFVELARE DKAAYVFYPH TIYKEYGQHM

                          301
NC_000964:1869011-1871071   DEMDMNEMYD KFVDNPRVKK EKINPRKLLE KLAMLRSESG YPYIMFQDNV
NC_CP01731:1840890-1842950  DEMDMNEMYD KFVDNPRVKK EKINPRKLLE KLAMLRSESG YPYIMFQDNV
NC_014976:4079508-4081568   DEMDMNEMYD KFVDNPRVKK EKINPRKLLE KLAMLRSESG YPYIMFQDNV
NC_022898:1832049-1834109   DEMDMNEMYD KFVDNPRVKK EKINPRKLLE KLAMLRSESG YPYIMFQDNV
NC_018520:1848510-1850570   DEMDMNEMYD KFVDNPRVKK EKINPRKLLE KLAMLRSESG YPYIMFQDNV
NC_017195:1843504-1845564   DEMDMNEMYD KFVDNPRVKK EKINPRKLLE KLAMLRSESG YPYIMFQDNV
NC_014479:1826454-1828514   DEMDMNEMYD KFVDNPRVKK EKINPRKLLE KLAMLRSESG YPYIMFQDNV

                          351
NC_000964:1869011-1871071   NKVHANNHIS KVKFSNLCSE VLQASQVSSY TDYDEEDEIG LDISCNLGSL
NC_CP01731:1840890-1842950  NKVHANNHIS KVKFSNLCSE VLQASQVSSY TDYDEEDEIG LDISCNLGSL
NC_014976:4079508-4081568   NKVHANNHIS KVKFSNLCSE VLQASQVSSY TDYDEEDEIG LDISCNLGSL
NC_022898:1832049-1834109   NKVHANNHIS KVKFSNLCSE VLQASQVSSY TDYDEEDEIG LDISCNLGSL
NC_018520:1848510-1850570   NKVHANNHIS KVKFSNLCSE VLQASQVSSY TDYDEEDEIG LDISCNLGSL
NC_017195:1843504-1845564   NKVHANNHIS KVKFSNLCSE VLQASQVSSY TDYDEEDEIG LDISCNLGSL
NC_014479:1826454-1828514   NKVHANNHIS KVKFSNLCSE VLQASQVSSY TDYDVEDEIG LDISCNLGSL
```

Figure S12: Visualization of a portion of the protein alignment of *B. subtilis* genes with high numbers of substitutions. Alignment visualization was performed with `SeaView` (Gouy et al. 2010)

High Substitution Distribution

Bacteria and Replicon	Bidirectional Genomic Position (bp)	Protein/Gene Examples
E. coli Chromosome	1130000 - 1140000	Uncharacterized proteins
		Hypothetical proteins
		Lipoprotein
		Transcriptional activator
	1720000 - 1740000	Hypothetical proteins
		Predicted protein
		Small toxic polypeptide
B. subtilis Chromosome	1990000 - 2000000	Hypothetical proteins
		Unknown function
Streptomyces Chromosome	3550000 - 3570000	Hypothetical proteins
		Derived by automated computational analysis
		Putative integral membrane protein
		Reductase
S. meliloti Chromosome	180000 - 200000	Hypothetical proteins
		Small molecule metabolism
S. meliloti pSymA	790000 - 800000	Hypothetical proteins
		Transposase
		Small molecule metabolism
S. meliloti pSymB	610000 - 620000	Hypothetical proteins
		Transposon related functions
		Predicted membrane protein

Table S10: Table of high number of substitutions per 10Kbp genomic regions for each bacterial replicon and examples of the associated proteins/gene functions found in that region. The genomic position begins at the origin of replication and continues in both directions until the terminus of replication (bidirectional replication).

Weighted, Non-weighted, and 20Kbp Near and Far From the Origin Substitution Linear Regression Analysis

Multiple linear regressions were performed to determine if there was any correlation between number of substitutions and distance from the origin of replication. A linear regression to determine how the weighted and non-weighted total number of substitutions in various sections of the genome (10Kbp, 25Kbp, 50Kbp, 100Kbp, 200Kbp, and 400Kbp) changes with genomic position was performed (Tables S11 and S12). All additional linear regression results (Tables S11 and S12) mirror the results from the logistic regression on presence or absence of substitutions and changes in genomic position (see the Main Paper results section for more information). The results from these supplemental tests are consistent with the results from the linear regression found in the Main Paper, most bacterial replicons have a decreasing number of substitutions when moving away from the origin of replication.

To calculate the non-weighted values of the total number of substitutions per 10Kbp region of the genome, the total number of substitutions was summed up over each region of the genome (10Kbp, 25Kbp, 50Kbp, 100Kbp, 200Kbp, and 400Kbp), while accounting for bidirectional replication (see Main Paper for details). A linear regression on these total number of substitutions in each section of the genome (10Kbp, 25Kbp, 50Kbp, 100Kbp, 200Kbp, and 400Kbp) was performed to see how the number of substitutions changes with distance from the origin of replication (Table S12). The weighted values of the total number of substitutions per various region of the genome, the total number of substitutions was summed up over each region of the genome (10Kbp, 25Kbp, 50Kbp, 100Kbp, 200Kbp, and 400Kbp) while accounting for bidirectional replication (see Main Paper for details). These summed values were then divided by the total number of protein coding sites in each region to obtain the weighted value. A linear regression on these weighted total number of substitutions in each section of the genome was performed to see how the number of substitutions changes with distance from the origin of replication (Table S11).

The non-significant (NS) linear regression results from Tables S12 and S11 are likely due to a decrease in the number of data points due to the nature of the methods for this supplemental analysis. In the windowed analysis (Tables S12 and S11) the total number of substitutions per various window size (10Kbp, 25Kbp, 50Kbp, 100Kbp, 200Kbp, and 400Kbp) were summed. This reduces the total number of data points used in the linear regressions, resulting in non-significant (NS) coefficient estimates. For example, the replicon of pSymA in *S. meliloti* only has a total length of 1.63Mbp and roughly 16.3 million data points including all ancestral and extant substitutions/genomic positions. When the total number of substitutions is summed over each region of the genome, these data points are collapsed to summarize what is happening in each local window. Lets take the 400Kbp window for example, when the total number of substitutions is summed over each 400Kbp region of the genome, the number of data points is drastically reduced to about 40. It is therefore unlikely that 40 data points provide enough information to detect a significant trend between the number of substitutions and distance from the origin of replication. This same logic can be applied to the other bacteria and window sizes. We therefore conclude that the lack of detection of a significant trend (NS) in Tables S12 and S11 is due to the decreased number of data points.

We took a closer look at 20Kbp regions of the replicons close and far from the origin of replication. We performed a logistic regression on the presence or absence of a substitution with distance from the origin of replication. Data points from the 20Kbp regions closest to the origin of replication and data points from the 20Kbp regions closest to the terminus of replication were used for this portion of the analysis. Outliers were removed from this analysis. The number of substitutions per site was also calculated in each of these 20Kbp regions for each bacterial replicon. We were unable to determine a consistent spatial substitution trend when considering only the 20Kbp near and far from the origin of replication in all bacterial replicons. Some bacterial replicons had a positive correlation coefficient, indicating that the number of substitutions increases with increasing distance from the origin of replication (Table S13). Other replicons had a negative correlation coefficient, suggesting that the number of substitutions decreases with increasing distance from the origin of replication (Table S13). Additionally, it was unclear if the number of substitutions per site locally were higher near the origin of replication or near the terminus. Some bacteria had higher number of substitutions per site near the origin (*Streptomyces*, *S. meliloti* chromosome and pSymB), while other replicons has the opposite trend (*E. coli*, *B. subtilis*, and *S. meliloti* pSymA) (Table S13). These results suggest that on a small local scale, there are varying patterns of substitutions with respect to distance from the origin of replication. This varies between bacteria, and in some cases even within the same bacteria (*S. meliloti* pSymB). This variation locally does not allow us to make any overarching statements about the local distribution of substitutions in bacterial genomes. It is therefore more useful to consider the global (genome wide) pattern of substitutions when making overarching statements about genomic substitution arrangements.

	Protein Coding Window Size					
Bacteria and Replicon	10Kbp	25Kbp	50Kbp	100Kbp	200Kbp	400Kbp
E. coli Chromosome	-2.27×10^{-10}*** (0.038)	-2.54×10^{-10}** (0.078)	-2.32×10^{-10}** (0.112)	-2.36×10^{-10}* (0.133)	NS (0.200)	NS (0.362)
B. subtilis Chromosome	NS (0.009)	NS (0.001)	NS (0.0002)	NS (0.002)	NS (0.019)	NS (0.484)
Streptomyces Chromosome	NS (2.49×10^{-5})	NS (2.12×10^{-5})	NS (0.004)	NS (0.0002)	3.68×10^{-11}* (0.126)	NS (0.182)
S. meliloti Chromosome	-1.21×10^{-10}** (0.076)	-1.71×10^{-10}*** (0.137)	-1.86×10^{-10}** (0.126)	-2.78×10^{-10}** (0.350)	NS (0.150)	NS (0.397)
S. meliloti pSymA	NS (0.032)	NS (0019)	NS (0.135)	NS (0.0124)	NS (0.034)	NS (1.42×10^{-30})
S. meliloti pSymB	NS (0.001)	NS (0.003)	NS (0.008)	NS (0.006)	NS (2.12×10^{-8})	NS (0.043)

Table S11: Linear regression on various sections of the genome (10Kbp, 25Kbp, 50Kbp, 100Kbp, 200Kbp, and 400Kbp) with increasing distance from the origin of replication after accounting for bidirectional replication. The total number of substitutions in each section of the genome was divided by the total number of protein coding sites in that genomic region (weighted). All results are marked with significance codes as followed: < 0.001 = '***', $0.001 < 0.01$ = '**', $0.01 < 0.05$ = '*', > 0.05 = 'NS'. The R^2 value for each coefficient estimate is found below the value in brackets ().

	Protein Coding Window Size					
Bacteria and Replicon	10Kbp	25Kbp	50Kbp	100Kbp	200Kbp	400Kbp
E. coli Chromosome	-1.66×10^{-4}*** (0.398)	-4.12×10^{-4}*** (0.476)	-8.64×10^{-4}*** (0.563)	-1.71×10^{-3}*** (0.509)	-3.42×10^{-3}** (0.534)	-6.71×10^{-3}* (0.592)
B. subtilis Chromosome	NS (0.004)	NS (0.004)	NS (0.001)	NS (0.001)	NS (0.145)	NS (0.027)
Streptomyces Chromosome	NS (0.002)	NS (0.007)	NS (0.014)	NS (0.025)	NS (0.073)	NS (0.074)
S. meliloti Chromosome	-8.97×10^{-6}*** (0.040)	-3.72×10^{-5}** (0.098)	-7.76×10^{-5}* (0.126)	-1.64×10^{-4}* (0.188)	NS (0.082)	NS (0.427)
S. meliloti pSymA	NS (0.027)	NS (0.001)	NS (0.006)	NS (0.193)	NS (0.050)	NS (1.59×10^{-31})
S. meliloti pSymB	NS (0.035)	NS (0.053)	NS (0.010)	NS (0.002)	NS (0.495)	NS (0.491)

Table S12: Linear regression on various sections of the genome (10Kbp, 25Kbp, 50Kbp, 100Kbp, 200Kbp, and 400Kbp) with increasing distance from the origin of replication after accounting for bidirectional replication. The linear regression was performed on the total number of substitutions in each section of the genome without accounting for the number of sites in each genomic region (non-weighted). All results are marked with significance codes as followed: < 0.001 = '***', $0.001 < 0.01$ = '**', $0.01 < 0.05$ = '*', > 0.05 = 'NS'. The R^2 value for each coefficient estimate is found below the value in brackets ().

	Protein Coding			
	Correlation Coefficient 20kb Near		Number of Substitutions per 20kb Near	
Bacteria and Replicon	Origin	Terminus	Origin	Terminus
E. coli Chromosome	NS	6.16×10^{-6}**	5.85×10^{-3}	6.47×10^{-3}
B. subtilis Chromosome	1.18×10^{-6}*	1.57×10^{-5}***	4.23×10^{-3}	5.01×10^{-3}
Streptomyces Chromosome	NS	NS	2.36×10^{-4}	2.05×10^{-5}
S. meliloti Chromosome	7.11×10^{-6}***	NS	1.51×10^{-3}	3.86×10^{-5}
S. meliloti pSymA	-6.94×10^{-5}***	NS	2.03×10^{-3}	3.27×10^{-3}
S. meliloti pSymB	1.58×10^{-5}***	-7.10×10^{-5}***	3.06×10^{-3}	1.25×10^{-3}

Table S13: Logistic regression on 20kb closest and farthest from the origin of replication after accounting for bidirectional replication and outliers. Number of substitutions was calculated by taking the total number of substitutions in each of the 20Kbp regions and dividing by the total number of sites in those regions. All results are marked with significance codes as followed: < 0.001 = '***', $0.001 < 0.01$ = '**', $0.01 < 0.05$ = '*', > 0.05 = 'NS'.

Non-linear Analysis of Number of Substitutions and Distance From the Origin of Replication

Using a simple smoothed conditional means method (`geom_smooth()` function in `R`), a non-linear trend analysis was performed on all bacterial replicons. The previous mentioned weighted data (see the previous subsection), was used in this analysis. The weighted data represents the total number of substitutions divided by the total number of protein-coding sites in 10Kbp segments of the genomes. Outliers were removed. The results from this non-linear analysis can be seen in Figures S13 - S18. The visual results from this analysis mirror the findings from the main

paper, the total number of substitutions varies with distance from the origin of replication, but the direction of this trend is unclear and inconsistent between bacterial replicons.

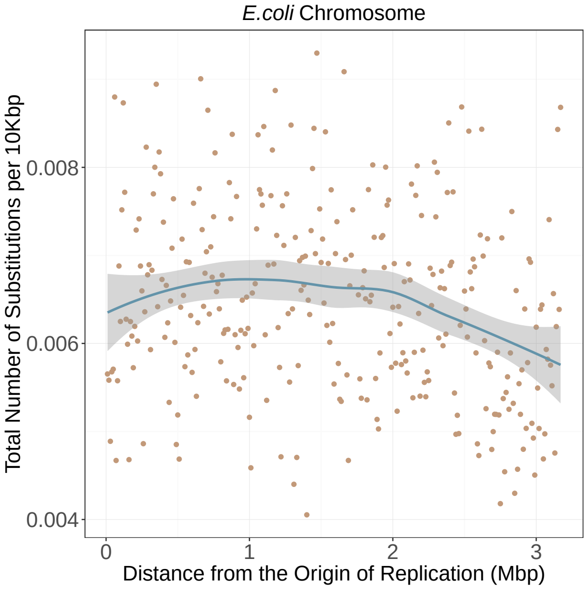

Figure S13: The graph shows the total number of substitutions weighted by the total number of protein-coding sites per 10Kbp segments of the *E. coli* genome. Each of these individual values are represented by beige coloured circles. A non-linear trend line (using the `geom_smooth()` function in `R`), was fit to these average values and the associated 95% confidence intervals for this line is represented by the grey ribbon around the blue trend line. Outliers were removed from this graph.

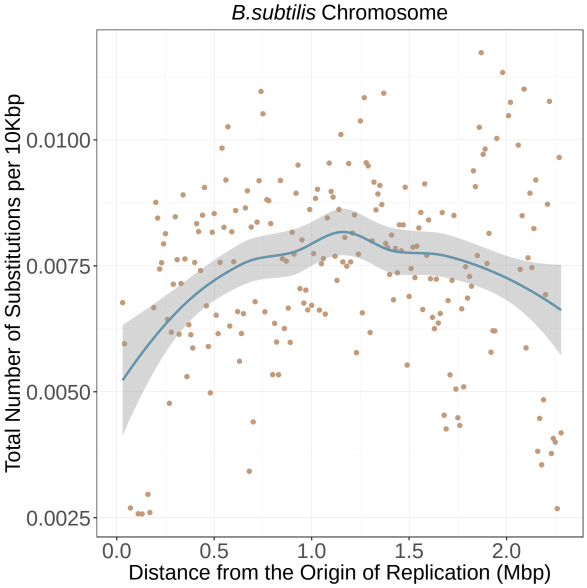

Figure S14: The graph shows the total number of substitutions weighted by the total number of protein-coding sites per 10Kbp segments of the *B. subtilis* genome. Each of these individual values are represented by beige coloured circles. A non-linear trend line (using the `geom_smooth()` function in `R`), was fit to these average values and the associated 95% confidence intervals for this line is represented by the grey ribbon around the blue trend line. Outliers were removed from this graph.

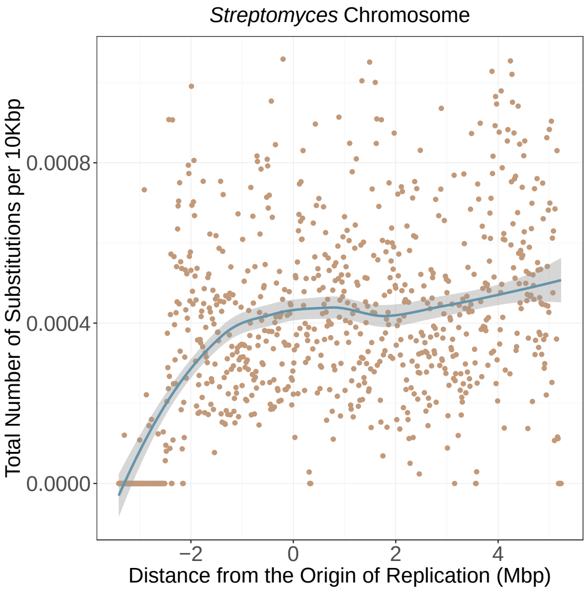

Figure S15: The graph shows the total number of substitutions weighted by the total number of protein-coding sites per 10Kbp segments of the *Streptomyces* genome. Each of these individual values are represented by beige coloured circles. A non-linear trend line (using the `geom_smooth()` function in `R`), was fit to these average values and the associated 95% confidence intervals for this line is represented by the grey ribbon around the blue trend line. Outliers were removed from this graph.

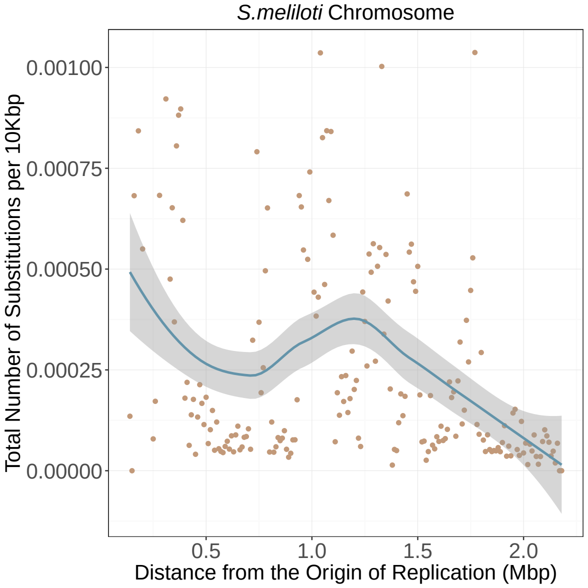

Figure S16: The graph shows the total number of substitutions weighted by the total number of protein-coding sites per 10Kbp segments of the *S. meliloti* Chromosome. Each of these individual values are represented by beige coloured circles. A non-linear trend line (using the `geom_smooth()` function in `R`), was fit to these average values and the associated 95% confidence intervals for this line is represented by the grey ribbon around the blue trend line. Outliers were removed from this graph.

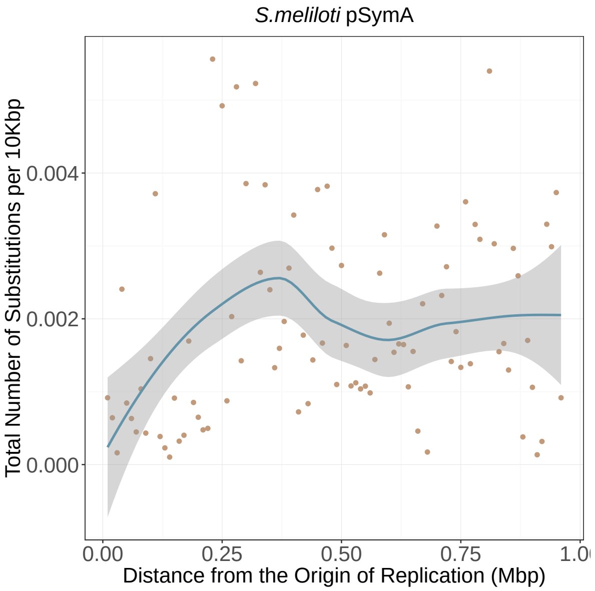

Figure S17: The graph shows the total number of substitutions weighted by the total number of protein-coding sites per 10Kbp segments of the *S. meliloti* pSymA replicon. Each of these individual values are represented by beige coloured circles. A non-linear trend line (using the `geom_smooth()` function in `R`), was fit to these average values and the associated 95% confidence intervals for this line is represented by the grey ribbon around the blue trend line. Outliers were removed from this graph.

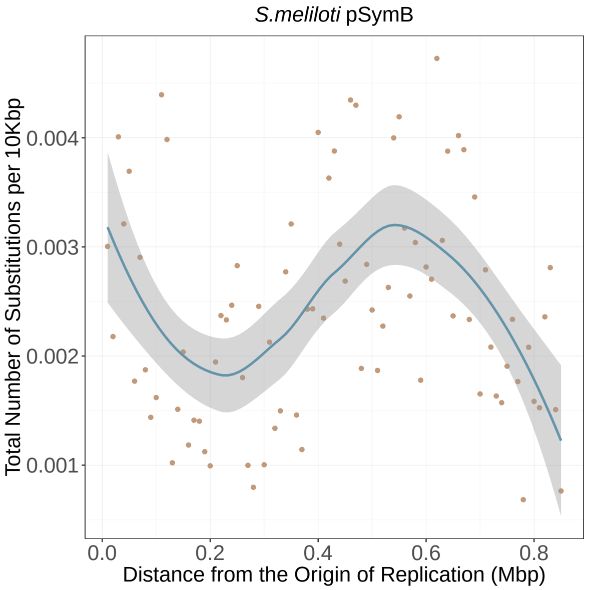

Figure S18: The graph shows the total number of substitutions weighted by the total number of protein-coding sites per 10Kbp segments of the *S. meliloti* pSymB replicon. Each of these individual values are represented by beige coloured circles. A non-linear trend line (using the `geom_smooth()` function in `R`), was fit to these average values and the associated 95% confidence intervals for this line is represented by the grey ribbon around the blue trend line. Outliers were removed from this graph.

Total Number of Sites Linear Regression

We performed a linear regression on the total number of protein coding sites and distance from the origin of replication (Table S14). We found that the total number protein coding sites decreases with distance from the origin of replication in majority of the bacterial replicons in this analysis. We were unable to detect a significant relationship between the number of protein coding sites and distance from the origin of replication in *B. subtilis*, the chromosome, and pSymB of *S. meliloti*.

Bacteria and Replicon	Coefficient Estimate	R^2
E. coli Chromosome	-2.33×10^{-2}***	0.423
B. subtilis Chromosome	NS	0.001
Streptomyces Chromosome	-4.09×10^{-3}***	0.095
S. meliloti Chromosome	NS	0.013
S. meliloti pSymA	NS	0.002
S. meliloti pSymB	2.69×10^{-2}**	0.081

Table S14: Linear regression analysis of the total number of protein coding sites per 10kb along the genome of the respective bacteria replicons. Linear regression was calculated after the origin of replication was moved to the beginning of the genome and all subsequent positions were scaled around the origin accounting for bidirectional replication. All results are marked with significance codes as followed: < 0.001 = '***', $0.001 < 0.01$ = '**', $0.01 < 0.05$ = '*', > 0.05 = 'NS'.

Robust "Leave One Out" Analysis on Substitution Data

Due to the computational and data availability limitations in the quantity of genomes chosen for each bacteria, we have performed an additional test to determine the robustness of our results. We have systematically removed/left out each taxa from the original substitutions analysis (as described in the Main Paper) this is a "**L**eave **O**ne **O**ut" (**LOO**) analysis. The goal of this analysis is to see if the overall results, that the number of substitutions significantly varies with distance from the origin of replication but the sign of this correlation is inconsistent, changes when any one taxa is removed. We want to ensure that our particular data sets are not influencing our conclusions. The original whole genome alignments specified by `progressiveMauve` and re-aligned with `MAFFT` following our various alignment quality criteria (see Methods) was used for this LOO analysis. The sequences from each taxa were systematically removed/left out from these alignment blocks. The original phylogenetic trees and corresponding branch lengths (Figures S4 - S9) were altered so that the same taxa that was remove from the alignment blocks was also removed from the phylogenetic tree, while maintaining correct branch lengths. These LOO alignment blocks and trees were then subject to the same methods for the substitution analysis (see Methods in Main Paper) where the ancestral nucleotide and genomic position was determined for each protein-coding site in the alignment blocks. A logistic regression was performed to determine the relationship between the number of substitutions and distance from the origin of replication (see Methods). A summary of these logistic regression results with each taxa removed can be found in Table S15.

The results from the chromosomes of *E. coli* and *S. meliloti* (Table S15) indicate that removing any of the taxa from these analysis, results in the same overall conclusion, that the number of substitutions decreases with increasing distance from the origin of replication. For the remaining replicons (*B. subtilis*, *Streptomyces*, pSymA and pSymB of *S. meliloti*), majority of the LOO results mirror what was found in the main paper when all taxa were present in the analysis (Table S15). However, there are some specific taxa that cause a reversal in the sign of the coefficient estimate. In the case of *Streptomyces* and pSymA in *S. meliloti*, the taxa which causes a reversal in sign when removed (*S. lividans* 1362 CM001889 and *S. meliloti* BL225C NC_017324 respectively) alter the location of the "outgroup" on the phylogenetic trees (Figures S6 and S8 respectively). When *S. lividans* 1362 CM001889 is removed, the new outgroup for the phylogenetic tree (Figure S6) becomes *S. lividans* TK24. When pSymA from *S. meliloti* BL225C is

removed, the clade containing *S. meliloti* 2011 and 1021 is now in the outgroup position. This shift in the outgroup is the cause for the coefficient estimate changing sign when these particular taxa (*S. lividans* 1362 CM001889 and *S. meliloti* BL225C NC_017324) are removed from the analysis. For *B. subtilis* and pSymB of *S. meliloti*, the taxa which caused a reversal in sign when removed (*B. subtilis* BSn5 NC_014976 and *S. meliloti* RMO17 CP009146) are located more in the inner parts of the phylogentic trees (Figures S5 and S9). These particular taxa heavily influence the ancestral genomic positions present throughout the phylogenetic tree. When these particular taxa are removed, this changes the ancestral genomic positions and alters ancestrally where the substitutions are located. This then influences the distribution of substitutions along the replicons enough to cause a change in the sign of the coefficient estimate (Table S15).

Since most of the results from the LOO analysis are the same as what was found in the main paper for each respective replicon, we maintain that our findings are robust even with the systematic removal of each taxa. The number of substitutions significantly varies with distance from the origin of replication, but the sign of this correlation is inconsistent.

Taxa Removed	Coefficient Estimate
E. coli	
None	-2.66×10^{-8}***
U00096	-3.12×10^{-8}***
CP0032890	-3.07×10^{-8}***
CU9281640	-2.95×10^{-8}***
CP0018550	-1.50×10^{-8}***
BA0000070	-2.63×10^{-8}***
CU9281630	-2.49×10^{-8}***
B. subtilis	
None	2.76×10^{-8}***
NC_000964	2.96×10^{-8}***
NC_018520	3.57×10^{-8}***
NC_017195	1.00×10^{-7}***
NC_022898	5.17×10^{-8}***
NC_014976	-4.02×10^{-8}***
CP01731	5.43×10^{-8}***
NC_014479	NS
Streptomyces	
None	7.21×10^{-8}***
CP050522	8.37×10^{-8}***
GG657756	3.62×10^{-8}***
CP042324	7.72×10^{-8}***
AL645882	7.65×10^{-8}***
CM001889	-2.46×10^{-7}***

Taxa Removed	Coefficient Estimate
S. meliloti Chromosome	
None	-6.57×10^{-7}***
NC_015590	-3.18×10^{-7}***
NC_003047	-6.01×10^{-7}***
CP004140	-6.00×10^{-7}***
CP009144	-6.67×10^{-7}***
NC_017322	-7.19×10^{-7}***
NC_017325	-5.01×10^{-7}***
S. meliloti pSymA	
None	2.74×10^{-7}***
NC_017327	6.98×10^{-7}***
CP009145	1.78×10^{-7}***
NC_003037	2.09×10^{-7}***
CP004138	2.08×10^{-7}***
NC_015591	NS
NC_017324	-1.52×10^{-6}***
S. meliloti pSymB	
None	1.10×10^{-7}***
NC_015596	6.78×10^{-7}***
NC_017326	1.67×10^{-7}***
NC_017323	NS
CP009146	-2.57×10^{-7}***
CP004139	1.04×10^{-7}***
NC_003078	1.04×10^{-7}***

Table S15: Logistic regression on the presence or absence of a substitution and distance from the origin of replication. Each strain was systematically removed and the entire analysis was repeated. All results are marked with significance codes as followed: < 0.001 = '***', $0.001 < 0.01$ = '**', $0.01 < 0.05$ = '*', > 0.05 = 'NS'.

High *dS* Values

Throughout this analysis there are a few genes/gene segments in all the bacterial replicons that have relatively high *dS* values. This is particularly evident in *B. subtilis* near 0.5Mbp from the origin of replication. Although we have rigorous and conservative methods for our sequence alignment and trimming, there appear to be some genes that are well aligned and similar for portions of the gene, but are quite divergent for other regions of the same gene. To illustrate this, we have chosen a gene alignment from this high *dS* region in *B. subtilis* located around 0.5Mbp from the origin of replication. The genes in this alignment can be found in Table S16. A simple `Clustal Omega` protein alignment of these genes can be found below. In this example it is evident that some portions of the gene have almost 100% sequence identity, while others are drastically divergent. These divergent regions are typically have a length close to our minimum 100bp trimming length, and are retained in our analysis in some cases. These divergent regions are what is driving the high *dS* values in our analysis.

Species	NCBI Accession Number	Gene Id
B. subtilis 168 *	NC_000964	BSU12750
B. subtilis BS38	NZ_CP017314	BSBS38_RS07215
B. subtilis BSn5	NC_014976	BSN5_RS18735
B. subtilis PY79	NC_022898	U712_RS06655
B. subtilis QB928	NC_018520	B657_RS07025
B. subtilis RONN1	NC_017195	I33_RS06720
B. subtilis W23	NC_014479	BSUW23_RS06800

Table S16: Information about the example gene alignment from *B. subtilis* with a high *dS* value.

```
CLUSTAL O(1.2.4) multiple sequence alignment


NC_014479      MAYEEKTDWLPDDPINEDDVNRWEKGIKDAHTDLAAHKNDMNNPHNTTKAQVGLGNVDNV     60
NC_000964      MAYEEKTDWLPDDPINEDDVNRWEKGIKDAHTDLAAHKNDMNNPHNTTKAQIGLGNVDNV     60
NC_022898      MAYEEKTDWLPDDPINEDDVNRWEKGIKDAHTDLAAHKNDMNNPHNTTKAQIGLGNVDNV     60
NC_018520      MAYEEKTDWLPDDPINEDDVNRWEKGIKDAHTDLAAHKNDMNNPHNTTKAQIGLGNVDNV     60
NZ_CP017314    MAYEEKTDWLPDDPINEDDVNRWEKGIKDAHTDLAAHKNDMNNPHNTTKAQIGLGNVDNV     60
NC_014976      MAYEEKTDWLPDDPINEDDVNRWEKGIKDAHTDLAAHKNDMNNPHNTTKAQIGLGNVDNV     60
NC_017195      MAYEEKTDWLPDDPINEDDVNRWEKGIKDAHTDLAVHKNDMNNPHNTTKAQIGLGNVDNV     60
               ***********************************.***************:********

NC_014479      KQAAKKDFDQHDQDQVRHIAEEEREKWNGGQLSKITKDDGSVFITID-NGQDFNEVAAQQ     119
NC_000964      QQASKTEFNEHNHDSTRHITSVERDEWNAKETPAGAQYKADQ------------------     102
NC_022898      QQASKTEFNEHNHDSTRHITSVERDEWNAKETPAGAQYKADQ------------------     102
NC_018520      QQASKTEFNEHNHDSTRHITSVERDEWNAKETPAGAQYKADQ------------------     102
NZ_CP017314    QQAAKKDFDKHEQDQVRHITSTERENWNAKETPGEAQNKADQ------------------     102
NC_014976      QQAAKKDFEKHVNDGTIHITAAERSKWNNAQLSKISGDDGRVFYKSVTEITDYNDL----     116
NC_017195      QQAAKKDFDKHISDETIHISSSERTKWNNAQLTKLTDEKGKYLASIQN-GLDFHKIVEEL     119
               :**:*.:*::*  * . **:  ** :**  :    :  ..

NC_014479      KKSFTFYTVKTGLNTPPQPTKGIYLYSSENDGEAIAMTNDGG-----IWR-KTLTSGEWS     173
NC_000964      ----------A---------------------EANAKAYTD------NFAAR--------     117
NC_022898      ----------A---------------------EANAKAYTD------NFAAR--------     117
NC_018520      ----------A---------------------EANAKAYTD------NFAAR--------     117
NZ_CP017314    ----------A---------------------EANAKAYTD------SFAAR--------     117
NC_014976      TDTGMYLIYNDGLNGPGLNQCFLLVMSYKN--TLVQIAYDGIKGEQSFFRIRKNDSTTWT     174
NC_017195      GQTFFFYTDKTGINTPPFATRGL-YIGYKSYGEALAMDYEGG-----TWR-KSLNDSGWT     172
                                                       .       :  :

NC_014479      EWASFETEAGSKSKAAQ-------------------------------------------     190
NC_000964      -------------------------------------------RD---------------     119
NC_022898      -------------------------------------------RD---------------     119
NC_018520      -------------------------------------------RD---------------     119
NZ_CP017314    -------------------------------------------RD---------------     119
NC_014976      AWIESETTEGSQKKIDAHANKTDIHVTKSDKDKWNDSQLFKITQDNGLAKYCEDA--DFN     232
NC_017195      DWVQLETSEGAQFKVRSHEEKTEIHVNKSDKDKWNSGQLFKVTADNGTQKINLSSGSFYD     232


NC_014479      ------------------------------------------------------------     190
NC_000964      ------------------NPNQVT-KA-----Q-----------VGLGNV----------     134
NC_022898      ------------------NPNQVT-KA-----Q-----------VGLGNV----------     134
NC_018520      ------------------NPNQVT-KA-----Q-----------VGLGNV----------     134
NZ_CP017314    ------------------NPNQVT-KA-----Q-----------VGLGNV----------     134
NC_014976      TVIETGFYYMSGATTTLNAPVNN--NGYLMVYNFSTYAYQEYTSYSSSDTISTGRRKFMR     290
NC_017195      SLKDVGTVTFYGTNAVTDNPSNTSLRGMQLVGQLG---------IGMGYAVDVGGNAWWF     283


NC_014479      ----------------AEKNAKNYIDNHTDNSSIHITNDERVKWNGAQLTKLTKDNGRRT     234
NC_000964      --------------ENVKQASLADFDAHLSNSKVHVSEGERNKWNAAQLIKLTGDDGKRI     180
NC_022898      --------------ENVKQASLADFDAHLSNSKVHVSEGERNKWNAAQLIKLTGDDGKRI     180
NC_018520      --------------ENVKQASLADFDAHLSNSKVHVSEGERNKWNAAQLIKLTGDDGKRI     180
NZ_CP017314    --------------ENVKQASQADFDAHLSNTKVHVSEGERNKWNAAQLIKLTGDDGKRI     180
NC_014976      NKVANSDVWTSWREIESVEGSQIKVDAHANKTDIHVTTSDKDKWNNAQLYRLTDTQGCRT     350
NC_017195     -FYNANDSAINWYQIESITGAQSKIDAHANKTDIHVTTSDKDKWNNAQLYRLTDTQGCRT      342
```

```
                                    :   .* * .::.:*:: .:: *** *** :**  :* *

NC_014479       WVPDGTDILSLSTGFYYGVGKYVVNNPVDDDNAWYNYDVIE-GESGRKTIVAYQSFEVTM   293
NC_000964       QLQDGTDILTLSSGFYCAVGQSVVNNPVEGDAAWYNYDIVE-GGSGRKTIVAYQSWGSMM   239
NC_022898       QLQDGTDILTLSSGFYCAVGQSVVNNPVEGDAAWYNYDIVE-GGSGRKTIVAYQSWGSMM   239
NC_018520       QLQDGTDILTLSSGFYCAVGQSVVNNPVEGDAAWYNYDIVE-GGSGRKTIVAYQSWGSMM   239
NZ_CP017314     QLQDGTDILTLSSGFYCAVGQSVVNNPVEGDATWYNYDIVE-GGSGRKTIVAYQSWGSMM   239
NC_014976       KIPDGTDLLTLPSGFYYALGNVITNNPVSGDGSWYNYDVIETEGGGRKTILASRSYDGTF   410
NC_017195       KIPDGTDLLTLPSGFYYAVGNVIINNPVLGDGSWYNYDVIETGGGGRKTIFASRSFDGTF   402
                 : ****:*:* :*** .:*: : **** .* :*****::*   .*****.* :*:   :

NC_014479       WIGMVHTDGKFRGWKRLVTSEELNSENINKITDESLYQDAAYSGNNYPIGITTVAILQGS   353
NC_000964       WIGMVHTDGEFRGWKQIATTDFIDRVQTELDLH--------ENDKTNPHSVTK-------   284
NC_022898       WIGMVHTDGEFRGWKQIATTDFIDRVQTELDLH--------ENDKTNPHSVTK-------   284
NC_018520       WIGMVHTDGEFRGWKQIATTDFIDRVQTELDLH--------ENDKTNPHSVTK-------   284
NZ_CP017314     WIGMVHTDGKFRGWKQIATTDFIDRVQSELDIH--------KNDKTNPHSVTK-------   284
NC_014976       WTATIHTDGVFKGWNKIETE----------------------------------------   430
NC_017195       WMATIHTDGVFKGWNKIETE----------------------------------------   422
                * . :**** *:**::: *

NC_014479       TGYPYELGEVLNIKSSKYRFAQFFFYAGNTGQKKVFIRHWYDTVGWTDFITIPSSEELES   413
NC_000964       --QQVGLGNVENVKQETPDGAQ--------KKADTALNQSKDYTNSTAFITRPLNS----   330
NC_022898       --QQVGLGNVENVKQETPDGAQ--------KKADTALNQSKDYTNSTAFITRPLNS----   330
NC_018520       --QQVGLGNVENVKQETPDGAQ--------KKADTALNQSKDYTNSTAFITRPLNS----   330
NZ_CP017314     --QQVGLGNVENVKQETPDGAQ--------KKADTALNQSKDYTNSTAFITRPLNS----   330
NC_014976       ------------------------------------------------------------   430
NC_017195       ------------------------------------------------------------   422


NC_014479       VLNTAKLYTDSHANNTEIHVTQNDKTKWNNSQIFKLTQDDGTLGKFYNEDLNNITKTGFY   473
NC_000964       ITDANDL---------------------------NLP--PGT----YRLDTNYMNAN---   354
NC_022898       ITDANDL---------------------------NLP--PGT----YRLDTNYMNAN---   354
NC_018520       ITDANDL---------------------------NLP--PGT----YRLDTNYMNAN---   354
NZ_CP017314     ITDANDL---------------------------NLP--PGT----YRLDTNYMNAN---   354
NC_014976       ------------------------------------------------------------   430
NC_017195       ------------------------------------------------------------   422


NC_014479       YIYSSTTELNAPINRNGYLLVYNVETYPYQEFTSYSGYTDSIPDNRRKFIRNKKQDSEEW   533
NC_000964       --PVLQNQFPLNDNRTGLLIIYPSANK-------WATRQDWFSISTKTLYTRVAVNGTDY   405
NC_022898       --PVLQNQFPLNDNRTGLLIIYPSANK-------WATRQDWFSISTKTLYTRVAVNGTDY   405
NC_018520       --PVLQNQFPLNDNRTGLLIIYPSANK-------WATRQDWFSISTKTLYTRVAVNGTDY   405
NZ_CP017314     --PELQNQFPLNDNRTGLLIIYPSANK-------WATRQDWFSISTKTLYTRVAVNGTEY   405
NC_014976       ------------------------------------------------------------   430
NC_017195       ------------------------------------------------------------   422


NC_014479       TPWMEIEYSQGAQAKADKALADAKNYVDTNYTNQKLTKLTGSNAIQDARTGGDEYPQGLT   593
NC_000964       SGWYILENSEGSQNKADKALADAKNYVETNYTNQKLTVLTGSNAIQDARISGNDYKYGIT   465
NC_022898       SGWYILENSEGSQNKADKALADAKNYVETNYTNQKLTVLTGSNAIQDARISGNDYKYGIT   465
NC_018520       SGWYILENSEGSQNKADKALADAKNYVETNYTNQKLTVLTGSNAIQDARISGNDYKYGIT   465
NZ_CP017314     TDWYILETSEGSQSKADKALADAKNYVDSNYTNNKLTVLTGSNAIQDARTSGNEYPAGLT   465
NC_014976       ---------VSAQTKADKALADAKNYVDSNYTNNKLTVLTGSNAIQDARTGGNEYPPGLT   481
NC_017195       ---------ASAQTKADKALSDAKNYVETNYTNQKLTVLTGSNAIQDARISGNDYKYGIT   473
                          .:* ******:******::****:*** *********** .*::*  *:*
```

```
NC_014479      LIDIGQGNNTGYPLRYGFVKNEKYSDFRFAQYFYGTGNESGSYIDSTGTWIRHWWSGSGW     653
NC_000964      FMDIGANNTTGYPLTYGFVKNEKHSNYRFTQYFYGNADTTSGSYDHVGTWIRHWWADSGW     525
NC_022898      FMDIGANNTTGYPLTYGFVKNEKHSNYRFTQYFYGNADTTSGSYDHVGTWIRHWWADSGW     525
NC_018520      FMDIGANNTTGYPLTYGFVKNEKHSNYRFTQYFYGNADTTSGSYDHVGTWIRHWWADSGW     525
NZ_CP017314    FMDIGANNTTGYPLTYGIVKNEKYSNYRFAQYFYGTGNESNSYFTSTGSWIRHWWSDSGW     525
NC_014976      LMDIGQGNTTGYPLGYGIVKNEKYSDFRFTQYFYGTGNESNSYIDSTGTWVRHWWSGSGW     541
NC_017195      FMDIGANNTTGYPLTYGFVKNEKHSNYRFTQYFYGNADTTSGSYDHVGTWIRHWWADSGW     533
               ::*** .*.***** **:*****:*::**:*****..: :..    .*:*:****:.***

NC_014479      TAWHKISGFAHAYIRTTGIQYLDKAAHTKIQFNRKIKDSHNAFDTKNSRFVAPNDGMFLV     713
NC_000964      TAWQKISGFAHANIGTTGRQALIKGENNKIKYNRIIKDSHKLFDTKNNRFVASHAGMHLV     585
NC_022898      TAWQKISGFAHANIGTTGRQALIKGENNKIKYNRIIKDSHKLFDTKNNRFVASHAGMHLV     585
NC_018520      TAWQKISGFAHANIGTTGRQALIKGENNKIKYNRIIKDSHKLFDTKNNRFVASHAGMHLV     585
NZ_CP017314    TAWHKISGFAHANIGTTGKQQLIKGELQKVKYNRKIKDSHNTFDTKNNRFIVPNDGMFLV     585
NC_014976      TAWQKISGFAHANIGTTGRQALIKGENNKIKYNRIIKDSHKLFDTKNNRFVASHAGMHLV     601
NC_017195      TAWQKISGFAHANIGTTGRQALIKGENNKIKYNRIIKDSHKLFDAKNNRFVASHAGMHLV     593
               ***:******** * *** * * *.   *:::** *****: **:**.**:. : **.**

NC_014479      GVGLYMINTPAYINFHLKLYLNGSLYKPIDHKRGDFV--DKENEMNLDLNGNVTVPMNKG     771
NC_000964      SASLYIENTERYSNFELYVYVNGTKYKLMNQFRMPTPSNNSDNEFNATVTGSVTVPLDAG     645
NC_022898      SASLYIENTERYSNFELYVYVNGTKYKLMNQFRMPTPSNNSDNEFNATVTGSVTVPLDAG     645
NC_018520      SASLYIENTERYSNFELYVYVNGTKYKLMNQFRMPTPSNNSDNEFNATVTGSVTVPLDAG     645
NZ_CP017314    NAGLYIENYQRYVNYELDIYLNGVRYKNIAHYRANPGDQSDTTEINVGLYGAATVPANQG     645
NC_014976      SASLYIENTERYSNFELYVYVNGTKYKLMNQFRMPTPSNNSDNEFNATVTGSVTVPLDAG     661
NC_017195      SASLYIENTERYSNFELYVYVNGTKYKLMNQFRMPTPSNNSDNEFNATVTGSVTVPLDAG     653
               ...**: *   * *:.* :*:**  ** : : *      .. .*:*  : * .*** : *

NC_014479      DYIEIYCYCNYHGTDRRGVSDYNEVYNYIDIQELGGLNYPTV       813
NC_000964      DYVEIYVYVGYSGDVTRYVTDSNGALNYFDVLELGGRNYPRV       687
NC_022898      DYVEIYVYVGYSGDVTRYVTDSNGALNYFDVLELGGRNYPRV       687
NC_018520      DYVEIYVYVGYSGDVTRYVTDSNGALNYFDVLELGGRNYPRV       687
NZ_CP017314    DYIEIYLYVGYNGGTTRYTTESSGWYNYFDITEIGGRNYPRT       687
NC_014976      DYVEIYVYVGYSGDVTRYVTDSNGALNYFDVLELGGRNYPRV       703
NC_017195      DYVEIYVYVGYSGDITRYVTDSNGVLNYFDVLELGGRNYPRV       695
               **:*** * .* *   * .:: .   **:*: *:** *** .
```

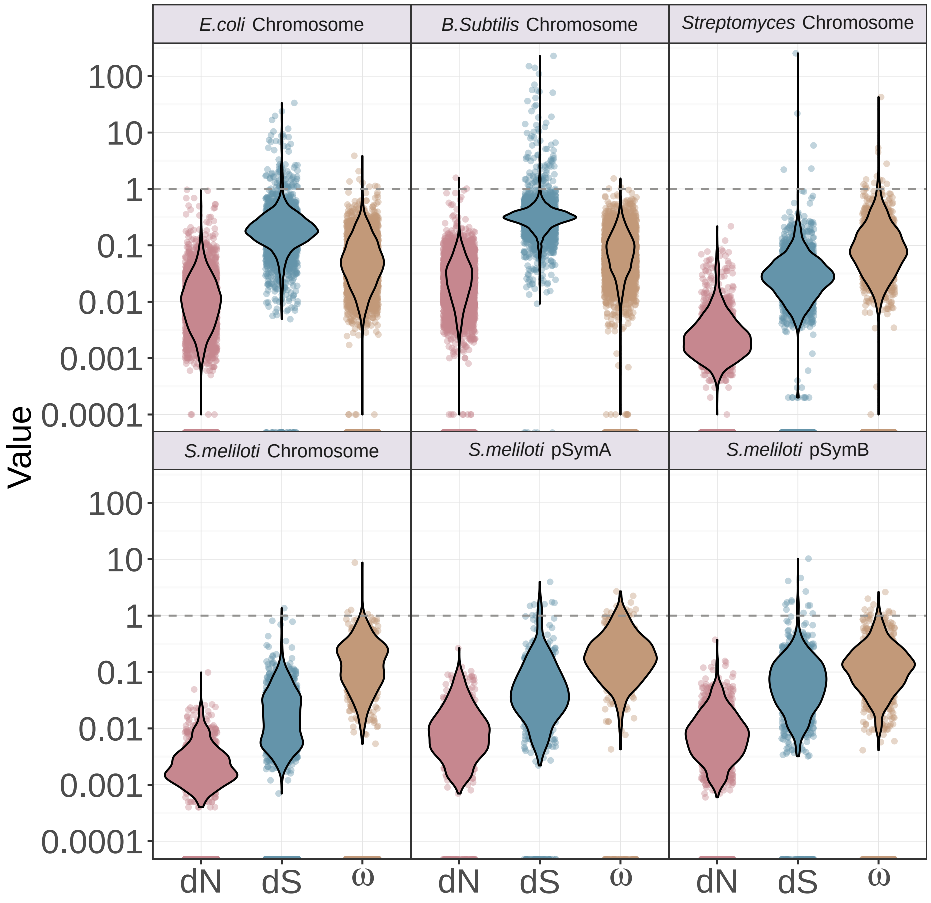

Figure S19: Distribution of all dN, dS, and ω values on a log base 10 scale for each replicon. Individual points are shown as a strip chart (which has been jittered in the x-direction in `R` (Wickham et al. 2019)), and the density of these selection values is shown in the overlaid violin plot. All points are included in this graphic including outliers. For more information on how outliers were calculated, please see the main paper. Any dN, dS, or ω values that had a value of zero is pushed to the bottom of the x-axis. Since these values will not appear on a log base 10 scale, they are not included in the violin portions of this graphic. For a complete list of zero values in each of the selection categories please refer to Table S17. In these graphs there is a horizontal line of values at 0.0001 for most of the selection coefficients in most of the bacterial replicons. This is due to rounding practices when `codeml` (Yang 1997) calculates dN, dS, and ω values.

Bacteria and Replicon	Outliers (%)	Zero Value (%)		
		dN	dS	ω
E. coli Chromosome	7.49	13.82	1.05	13.82
B. subtilis Chromosome	5.41	4.40	0.16	4.40
Streptomyces Chromosome	4.74	25.70	14.48	25.70
S. meliloti Chromosome	17.05	61.21	59.26	61.21
S. meliloti pSymA	6.69	11.28	9.75	11.28
S. meliloti pSymB	6.13	13.20	5.20	13.20

Table S17: Percent of data that was calculated to be an outlier or had a selection variable (dN, dS, and ω) value of zero.

Average dN, dS, and ω per Gene Values

The average dN, dS, and ω values per gene were calculated. For genes that were split into multiple parts (due to the presence of gaps or poor homology in the alignment), the dN, dS, and ω values for each gene part were averaged to obtain a single average value per gene. A complete list of these values can be found on `GitHub` (`www.github.com/dlato/Location_of_Substitutions_and_Bacterial_Arrangements`) under the file name "`Supplementary_table_per_gene_dN_dS_omega.pdf`".

Window Analysis for dN, dS, and ω

Multiple linear regressions were performed to determine if there was any correlation between the average dN, dS, and ω values and distance from the origin of replication. A linear regression to determine how the average dN, dS, and ω values in various sections of the genome (10Kbp, 25Kbp, 50Kbp, 100Kbp, 200Kbp, and 400Kbp) changes with genomic position was performed (Table S18). The results from these supplemental tests are consistent with the results from the linear regression found in the Main Paper, most bacterial replicons do not have a significant correlation between dN, dS, and ω values and distance from the origin of replication. Linear regressions that were significant, were inconsistent in sign.

20Kbp Near and Far From Origin Selection Linear Regression Analysis

We additionally took a closer look at 20 genes close and far from the origin of replication. We performed a linear regression on the change in selection values (dN, dS, and ω) with distance from the origin of replication in these genes (Table S19). For majority of the bacterial replicons we failed to find a trend, which is not surprising since there was no evidence of an overall genomic trend when looking at these values (see Main Paper for results). Again, we are unable to conclude that there is a consistent overall trend for any of the selection values, dN, dS, and ω.

	Near Origin			Near Terminus		
Bacteria and Replicon	dN	dS	ω	dN	dS	ω
E. coli Chromosome	NS	NS	NS	NS	NS	NS
B. subtilis Chromosome	NS	NS	NS	NS	NS	NS
Streptomyces Chromosome	NS	NS	-9.36×10^{-7}* (0.328)	NS	NS	NS
S. meliloti Chromosome	NS	NS	NS	NS	NS	NS
S. meliloti pSymA	NS	NS	NS	-2.53×10^{-7}* (0.238)	NS	NS
S. meliloti pSymB	NS	6.19×10^{-6}** (0.372)	NS	NS	4.92×10^{-6}* (0.232)	NS

Table S19: Linear regression for dN, dS, and ω calculated for each bacterial replicon for the 20 genes closest and 20 genes farthest from the origin of replication. All results are marked with significance codes as followed: p: < 0.001 = '***', $0.001 < 0.01$ = '**', $0.01 < 0.05$ = '*', > 0.05 = 'NS'. The R^2 values for each estimate are in brackets.

Bacteria and Replicon	Protein Coding Window Size 10Kbp	25Kbp	50Kbp	100Kbp	200Kbp	400Kbp
			dS			
E. coli Chromosome	NS (0.008)	NS (0.0168)	NS (0.0194)	NS (0.0332)	NS (0.0713)	NS (0.165)
B. subtilis Chromosome	NS (0.0057)	NS (0.0105)	NS (0.0198)	NS (0.0254)	NS (0.0743)	NS (0.113)
Streptomyces Chromosome	NS (0.002)	NS (0.00105)	NS (0.00139)	NS (0.00245)	NS (0.00401)	NS (0.00645)
S. meliloti Chromosome	NS (0.0143)	NS (0.0216)	NS (0.0293)	NS (0.0299)	NS (0.0676)	NS (0.111)
S. meliloti pSymA	NS (0.00775)	NS (0.0108)	NS (0.0177)	NS (0.0243)	NS (0.0315)	NS (0.912)
S. meliloti pSymB	NS (0.00582)	NS (0.0136)	NS (0.0164)	NS (0.0731)	NS (0.476)	NS (0.701)
			dN			
E. coli Chromosome	NS (0.0004)	NS (0.0001)	NS (0.0002)	-NS (0.0001)	NS (1.88×10^{-5})	NS (0.0132)
B. subtilis Chromosome	NS (0.0164)	NS (0.0365)	NS (0.0614)	NS (0.0685)	NS (0.127)	NS (0.15)
Streptomyces Chromosome	NS (0.00376)	NS (0.00196)	NS (0.00454)	NS (0.0005)	NS (0.00385)	NS (0.0154)
S. meliloti Chromosome	NS (0.0178)	NS (0.0213)	NS (0.0247)	NS (0.0245)	NS (0.0565)	NS (0.0836)
S. meliloti pSymA	NS (0.00671)	NS (0.00433)	NS (0.0128)	NS (0.0599)	NS (0.0329)	NS (0.736)
S. meliloti pSymB	NS (0.0001)	NS (2.4×10^{-6})	NS (0.0005)	NS (0.00311)	NS (0.128)	NS (0.24)
			ω			
E. coli Chromosome	5.22×10^{-9}*** (0.061)	4.62×10^{-9}*** (0.11)	5.62×10^{-9}*** (0.174)	4.96×10^{-9}** (0.296)	4.8×10^{-9}* (0.363)	3.51×10^{-9}* (0.51)
B. subtilis Chromosome	NS (0.0084)	NS (0.0281)	NS (0.0348)	NS (0.0185)	NS (0.0255)	NS (0.0179)
Streptomyces Chromosome	2.12×10^{-9}** (0.0104)	NS (0.0115)	1.98×10^{-9}* (0.0312)	NS (0.0308)	NS (0.0654)	NS (0.144)
S. meliloti Chromosome	-1.66×10^{-9}* (0.0278)	NS (0.0327)	NS (0.0337)	NS (0.0238)	NS (0.0383)	NS (0.0416)
S. meliloti pSymA	NS (0.00218)	NS (0.00326)	NS (0.00657)	NS (0.426)	NS (0.511)	NS (0.607)
S. meliloti pSymB	NS (0.0239)	NS (0.0662)	NS (0.098)	NS (0.002)	NS (0.634)	2.74×10^{-8}** (1)

Table S18: Linear regression on various sections of the genome (10Kbp, 25Kbp, 50Kbp, 100Kbp, 200Kbp, and 400Kbp) with increasing distance from the origin of replication after accounting for bidirectional replication. The linear regression was performed on the average dN, dS, and ω values in each section of the genome. All results are marked with significance codes as followed: < 0.001 = '***', $0.001 < 0.01$ = '**', $0.01 < 0.05$ = '*', > 0.05 = 'NS'. The R^2 value for each coefficient estimate is found below the value in brackets ().

Additional References

Capella-Gutiérrez S, Silla-Martinez J M, and Gabaldón T (2009). trimAl: a tool for automated alignment trimming in large-scale phylogenetic analyses. Bioinfor 25(15), 1972–1973.

Gouy M, Guindon S, and Gascuel O (2010). SeaView version 4: A multiplatform graphical user interface for sequence alignment and phylogenetic tree building. Mol Biol Evol 27, 221–224.

Wickham H, Averick M, Bryan J, Chang W, McGowan L D, François R, Grolemund G, Hayes A, Henry L, Hester J, et al. (2019). Welcome to the {tidyverse}. Journal of Open Source Software 4(43), 1686.

Yang Z (1997). PAML: a program package for phylogenetic analysis by maximum likelihood. Bioinfor 13(5), 555–556.
